# Bioinspired Magnetized String with Tension-Dependent Eigenfrequencies for Wearable Human–Machine Interactions

**DOI:** 10.1021/acsami.4c16653

**Published:** 2024-11-25

**Authors:** Biao Qi, Sen Ding, Yuanzhe Liang, Dan Fang, Ming Lei, Wenxue Dai, Chao Peng, Bingpu Zhou

**Affiliations:** † Joint Key Laboratory of the Ministry of Education, Institute of Applied Physics and Materials Engineering, University of Macau, Avenida da Universidade, Taipa, Macau 999078, P.R. China; ‡ School of Environmental and Chemical Engineering, Jiangmen Key Laboratory of Synthetic Chemistry and Cleaner Production and Institute of Carbon Peaking and Carbon Neutralization, Wuyi University, Jiangmen 529020, P.R. China; § Department of Physics and Chemistry, Faculty of Science and Technology, University of Macau, Avenida da Universidade, Taipa, Macau 999078, P.R. China

**Keywords:** human−machine interaction, eigenfrequency, tension, string vibration, flexible magnetized system

## Abstract

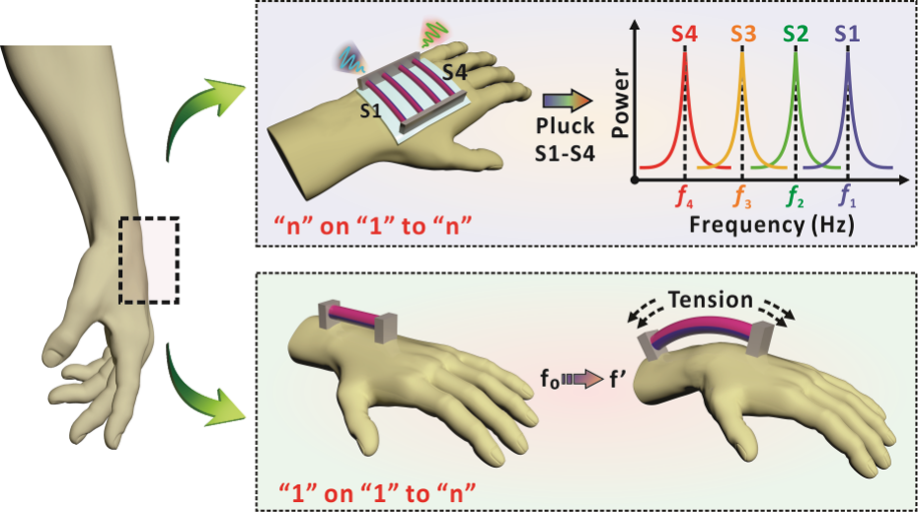

Flexible and wearable devices have exhibited potential for applications in the fields of human–machine interactions (HMIs) and Internet of Things. However, challenges remain in the improvement of the communication storage capacity with a simplified architecture. Inspired by tension regulation in natural tendons, a single-channel wearable HMI strategy is proposed using the eigenfrequency of magnetized strings as a sensing solution. Based on electromagnetic induction, mechanical vibration of the magnetized string can electrically induce periodical damping signals in the coil that are associated with the intrinsic eigenfrequency property of the string. Using a theoretical vibration model, nonoverlapping eigenfrequencies are precisely customized by designing the dimension/modulus or tension status of the string to broaden the eigenfrequency library. By integrating strings with different eigenfrequencies, multiple commands can be realized with a single communication channel. Moreover, identifiable commands can be flexibly tuned with only one magnetized string by customizing the tensile length (string tension) for eigenfrequency regulation. Demonstrations such as tactile addressing, authentication systems, and robotic control indicate the potential of the interface for multifunctional HMI applications. We expect that this strategy will provide a valuable reference for the future design of wearable HMI interfaces with high storage capacity and controllability in an accessible architecture.

## Introduction

Human–machine interaction (HMI) systems serve as a crucial link between humans and machines, playing significant roles in the field of virtual reality, robot control, the Internet of Things (IoT), etc.^1−8^ Recently, flexible sensors have been increasingly applied in HMI systems to convert the desires from human beings into signals that can be recognized by the electric terminal.^9^ Compared with rigid electronic devices, flexible sensors can better conform to the diverse shapes of the human body and also have the ability to change shape in sync with the body’s movements, thereby improving the natural and comfortable interaction in the HMI process. To date, various types of flexible sensors, such as resistive,^10^ capacitive,^11,12^ triboelectric,^13^ optical,^14^ and electromagnetic sensors,^15,16^ have been developed for sensing an extensive range of external stimuli, and they play a crucial role in situations where rigid sensors face limitations, exhibiting potential for real-time dynamic health monitoring and HMI in multiple scenarios.

Additionally, there has been a growing demand from society and users for more effective and realistic interaction experiences.^17^ This trend has drawn increasing attention to HMI systems with higher information storage and transmission capabilities.^18^ Typically, sensor arrays are a straightforward method to expand communication memory. However, more communication channels are required for flexible sensors to process the signals as the number of sensors increases, raising concerns about wiring redundancy and reduced portability.^19−21^ Therefore, it is crucial to increase the information-carrying capacity of individual devices and simplify the avenue for signal processing. Currently, researchers have proposed many strategies to improve the information capability of individual devices.^22−26^ For example, Wei et al. proposed an all-in-one multifunctional touch sensor composed of carbon-based gradient resistance elements, which enable multipoint touch control with only two electrodes by recognizing the change of resistance.^27^ Cao et al. reported a self-powered hybrid encoder based on the triboelectric nanogenerator, which combined single-electrode and contact-separation modes to recognize the two actions of finger touch and press and convert them into two different encoding formats.^28^ Lei et al. designed an electronic skin to distinguish the strain and pressure stimuli with positive and negative resistance changes for effective Morse code communication.^29^ However, challenges still exist if higher information storage is required for the HMI interface, and the concerns of power consumption, simple geometry, and wiring complexity are yet to be fully addressed.

Vibration, as a widespread phenomenon in nature, is a periodic motion produced by mechanical systems after perturbation from the equilibrium position. Moreover, eigenfrequency is the intrinsic vibration frequency of elastic systems such as cantilever beams, springs, strings, etc. and is not significantly affected by external triggering stimuli.^30,31^ Recent studies have demonstrated that the oscillation eigenfrequency of magnetized microstructures can be applied as the sensing mechanism for high-capacity HMI with a simplified architecture.^32,33^ Even though the principle allows communication to be processed in one signal channel, the fixed eigenfrequency related to the as-prepared geometry limits the controllability of the whole system. In view of this, we herein report a flexible wearable HMI interface based on the eigenfrequency of magnetized strings, which is inspired by a natural tendon that can easily tune the inherent tension via regulation of the tensile length. The magnetized strings can be fixed on the back of the hand or at the joints of the body, e.g., the wrist. Based on the principle of electromagnetic induction, mechanical vibration signals from the plucked strings can be transformed into electrical profiles with specific frequencies, which serve as the triggering signals to arouse corresponding commands in HMI applications. In the eigenfrequency mode, the output of commands is independent of the initial triggering stimuli, thus greatly improving the stability and accuracy of the communication process. The eigenfrequency of a magnetized string can be controlled by the dimension/modulus or tensile length of the string with a large adjustable range, which follows the classical two-end-fixed string vibration model. According to the target applications, the number of magnetized strings can be flexibly customized on the back of the hand, while only one coil layer and communication channel are required thanks to the nonoverlapping frequency values. Furthermore, the unique frequency of the stretchable magnetized strings can be easily adjusted by regulating the specific tension within the individual string. This behavior allows the production of multiple commands through one magnetized string via tension regulation such as pressing or moving with the joints, which greatly simplifies the overall configuration and increases the flexibility and functionality of the interaction interface. With the demonstrated accuracy and stability, such a magnetized string-based system can be applied as a promising interface for future HMI applications with high capacity and simple architecture.

## Experimental Section

### Materials

The polydimethylsiloxane (PDMS) base and curing agent (Sylgard 184 kit) were purchased from Dow Corning, USA. Ecoflex (model 00-50) was purchased from Smooth-On, Inc., USA. The neodymium–iron–boron (NdFeB) particles were obtained from Magnequench, China. The coil substrates were purchased from Chengdu Do-itc New Material Co., Ltd. (China) for electrode processing.

### Fabrication of Magnetized Strings

First, PDMS, Ecoflex, and NdFeB particles were uniformly mixed in different mass ratios and injected into a plastic mold, which was fabricated by a mechanical engraving machine (CNC 3020-300W, JingYan Instruments & Technology CO., LTD, China). The dimension, e.g., radius and length, of the patterns in the mold can be flexibly regulated based on the parameter setting during the engraving procedure. Before injection, a standard degassing process was applied to completely remove the air bubbles from the uncured composite. After injection, the sample was transferred to an oven at 80 °C and cured for 30 min to ensure complete solidification. Finally, the solidified PDMS/Ecoflex/NdFeB composites were peeled from the mold and placed horizontally in a commercial magnetization apparatus (MA-3030, Shenzhen Jiuju Industrial Equipment Co., Ltd.) to obtain magnetized strings. The magnetic field intensity for magnetization was ∼3 T.

### Fabrication of Coils and Assembly of Magnetized Strings

A laser engraving machine (LPKF ProtoLaser U4, LPKF Laser & Electronics AG, Germany) was used to ablate the double-layer copper coil on a copper electrode substrate. In the copper substrate, two copper layers with a thickness of 18 μm are separated by an insulating polyimide (PI) with a thickness of 75 μm. The width of each loop was 80 μm, and the distance between two adjacent conductive lines was 20 μm. The size of the coil can be controlled by changing the number of conductive loops. After laser ablation, the two layers of coils were electrically connected using commercial conductive silver paste. To ensure the stable assembly of magnetized strings, the two ends of the magnetized strings were firmly fixed by miniature screws to the polymeric block that was prepared by PDMS and NdFeB in the mass ratio of 1:5.

### Characterization

Scanning electron microscopy (SEM, Carl Zeiss, Germany) and energy-dispersive spectroscopy (EDS) were used to characterize the morphology and composition of the strings.

The magnetic field around the magnetized string was measured by a three-axis commercial Gauss-Tesla meter (DX-360, Dexin Mag, China). The current signals generated by magnetized string vibration in the coil were measured by a low-noise current preamplifier (MODEL SR570, SRS, USA) and a multifunctional I/O device (USB-6341, NI, USA). The elastic modulus of the material and the tensile force of the magnetized string under tension were measured by a commercial mechanical meter (ESM303, Mark-10 Corporation, USA). The vibration process of the magnetized string was recorded by a high-speed camera (VEO 710S, Phantom, USA) with a frame rate of 5000 frames per second. A laser Doppler vibrometer (LDV) was used to record the displacement and velocity of the magnetized string in real time during vibration. The wearable demonstrations were carried out by research volunteers with ensured safety during the characterization. Informed consents were obtained from the volunteers for related tests.

### Configuration Parameters for the LabVIEW Interface

The built-in DAQmx series subVIs in LabVIEW was used to configure the acquisition parameters of the signal including physical channels, sampling rate, sampling number, trigger conditions, etc. The sampling rate was set to 10,000 Hz. The sampling trigger condition was set to be >2 μA to prevent environmental noise from triggering the sampling. The sampling number was set to 1000, which is sufficient to extract the related eigenfrequency from the received signals. When the sampling was triggered, electric data within 0.1 s were transmitted to the fast Fourier transform (FFT) module for eigenfrequency extraction. Even though the string vibration continues after the first 100 ms, the related electric signals would normally be weak due to the damping effect. Consequently, this would not affect the following analysis if plucking of a magnetized string for subsequent eigenfrequency production is required in the demonstration.

### Simulation of Magnetic Field

The magnetic field around the magnetized string before and after deformation was simulated using the magnetic field (no current) module of COMSOL Multiphysics 6.1. The geometrical model of the magnetized string before and after deformation was imported by using the 3D print file format. The diameter of the magnetized string was 1.4 mm, the length was 30 mm, sintered NdFeB was used as the material of the magnetized string, and the air environment was set as a square with a side length of 100 mm. The governing equation for the magnetization model was *B* = μ_0_(*H* + *M*), where *B* is the magnetic flux density vector, *H* is the magnetic field vector, *M* is the magnetization vector, and μ_0_ is the permeability of vacuum. The boundary conditions were set as *n·B* = 0. The initial value of the magnetic scalar potential was set as 0.

### Simulation of the Eigenfrequency

COMSOL Multiphysics 6.1 was used to simulate the eigenfrequency of the magnetized strings with different diameters and tensile lengths. The magnetized string constructed in the built-in geometry module has an elastic modulus of 1.297 MPa, a density of 2013 kg/m^3^, and a Poisson ratio of 0.49. The eigenfrequency of the magnetized string was calculated using solid mechanics and eigenfrequency modules. In the solid mechanics module, fixed constraints were applied at both ends of the string, and an initial stress identical to the experimentally measured value was applied to the string for simulation.

## Results and Discussion

### Design and Working Principles

**Figure 1:**
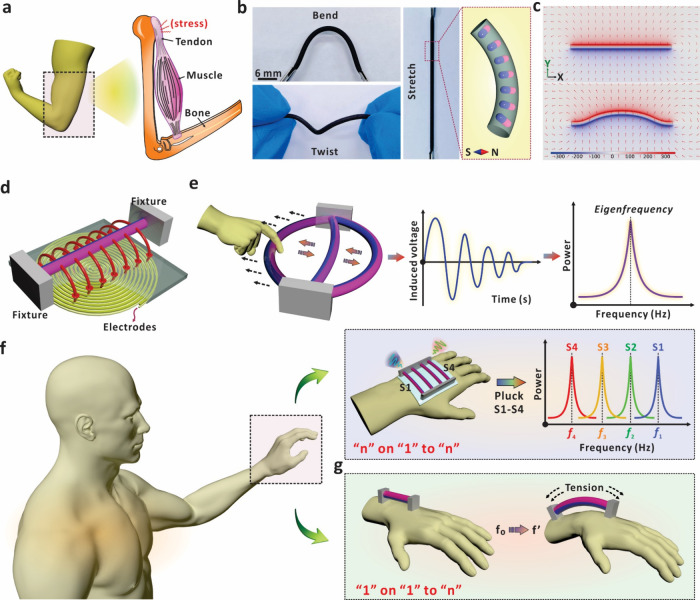
Design and working principle of magnetized strings for eigenfrequency-based HMI. (a) Schematic diagram of the human body structure during muscle contraction. (b) Optical images of the magnetized string in the stretched, bent, and twisted states. (c) Simulation results of the magnetic field around the magnetized string before and after deformation. The arrow volume is related to the magnetic flux density, and the slices indicate the magnetic scalar potential (unit: A). (d) Schematic diagram of the interface that contains a magnetized string and a coil. The two ends of the string were fixed on the coil layer. (e) Principle of the magnetized string for signal sensing and transmission. The induced current is generated when the magnetized string vibrates and the eigenfrequency is extracted by time-frequency conversion. (f) Schematic diagram of HMI based on multiple magnetized strings with customized eigenfrequencies. (g) Schematic diagram of HMI based on a single magnetized string via tension regulation.

As shown in Figure 1a, natural tendons act as a bridge between bones and muscles when humans contract muscles. The movement of joints in the human body relies on the stretching and contracting of tendons accompanied by an increase or decrease in the tension applied. As an important sensing component in the human body, the fibrous structure of tendons makes them highly resistant to mechanical loads and maintains their elasticity when sliding between muscles and bones. Inspired by this fixed structure at both ends, we designed magnetized strings that could also be easily stretched and contracted under tension in HMI systems, which serve as the sensing elements of mechanical stimuli and convert them into electrical signals that can be recognized by electrical terminals. As discussed below, the tunable tension within the string matrix can serve as a powerful parameter to customize the electric signals for command allocation in multifunctional HMI applications. Figure S1 shows the fabrication of the magnetized string that is composed of PDMS, Ecoflex, and NdFeB particles (see the Experimental Section for details). As shown by the SEM and EDS results in Figure S2, the NdFeB particles were uniformly distributed within the magnetized string matrix. After magnetization along the radial direction, the string could act as a flexible and stretchable magnet with well-defined south (S) and north (N) poles. The magnetized string has excellent flexibility to stretch, bend, and twist in response to external stimuli, and more importantly, it could be deformed or vibrated as a flexible magnet to generate signals thanks to the built-in magnetized orientation (Figure 1b). The simulation result in Figure 1c shows the variation of the localized magnetic flux intensity when the magnetized string was deformed. Furthermore, we applied a Gauss meter to measure the real-time magnetic field changes when a typical string was plucked. The results in Figure S3 indicate that at the moment of pluck and release, the deformation of the magnetized string can induce significant transient changes in the spatial magnetic field. Therefore, when the magnetized string vibrates, the bottom coil can sense the change of the magnetic field in the form of induced voltage according to Faraday’s law of induction (Figure 1d).^34,35^ The induced voltage can be described as , where *N* is the number of coil loops, dΦ is the change of magnetic flux, and d*t* is the corresponding duration. In this work, the flexible coil consists of two identical layers, and the separation between each conductive loop is about 20 μm to maximize the magnetic flux in the coil (Figure S4). The flexibility of the coil ensures its attachment on human skin for applications in a wearable HMI.

Figure 1e further demonstrates the basic principles of magnetized strings for signal sensing and transmission. Because of the excellent flexibility, the magnetized string could be easily deformed by human fingers. After releasing from the mechanical stimuli, the magnetized string would immediately initiate a damped vibration process due to the intrinsic recovery capability. Such vibration results in the spatial variation of the magnetic field, inducing electromotive force and the corresponding current in the bottom coil as mentioned above. Through time–frequency conversion of the electrical signals, the specific eigenfrequency of the string vibration can be obtained with a distinct peak in the frequency profile. As eigenfrequency is an inherent property of the magnetized string, the acquisition of eigenfrequency can thus serve as an identifiable cue to enable the allocation of specific commands for HMI applications. Based on this, we can flexibly adjust the parameters of the magnetized strings for eigenfrequency customization. For example, an interface with four magnetized strings of different eigenfrequencies can be established owing to the distinguishable frequency. When the specific string was plucked, a related eigenfrequency was transmitted to initiate the allocated command for further HMI applications (Figure 1f). Theoretically, “*n*” magnetized strings with identifiable eigenfrequencies can be assigned to “*n*” specific commands with only one shared communication channel. With “*n*” strings on “1” coil platform, “*n*” commands can be totally produced to enable an “*n*” on “1” to the “*n*” communication interface. In addition, the tension-dependent vibration frequency allows the allocation of multiple commands on a single magnetized string for more flexible HMI modes. As presented in Figure 1g, when the two ends of a magnetized string are fixed at the wrist joint of the human body, the bending of the wrist will lead to the corresponding change of the string length. This will finally result in the variation of the string tension and related eigenfrequency during the vibration. A “1” on “1” to “*n*” communication can thus be established as the allocation of “*n*” commands can be achieved with only one magnetized string on a “1” coil platform, which effectively simplifies the overall communication configuration for a more convenient HMI process.

### Characterization of the Magnetized String

**Figure 2:**
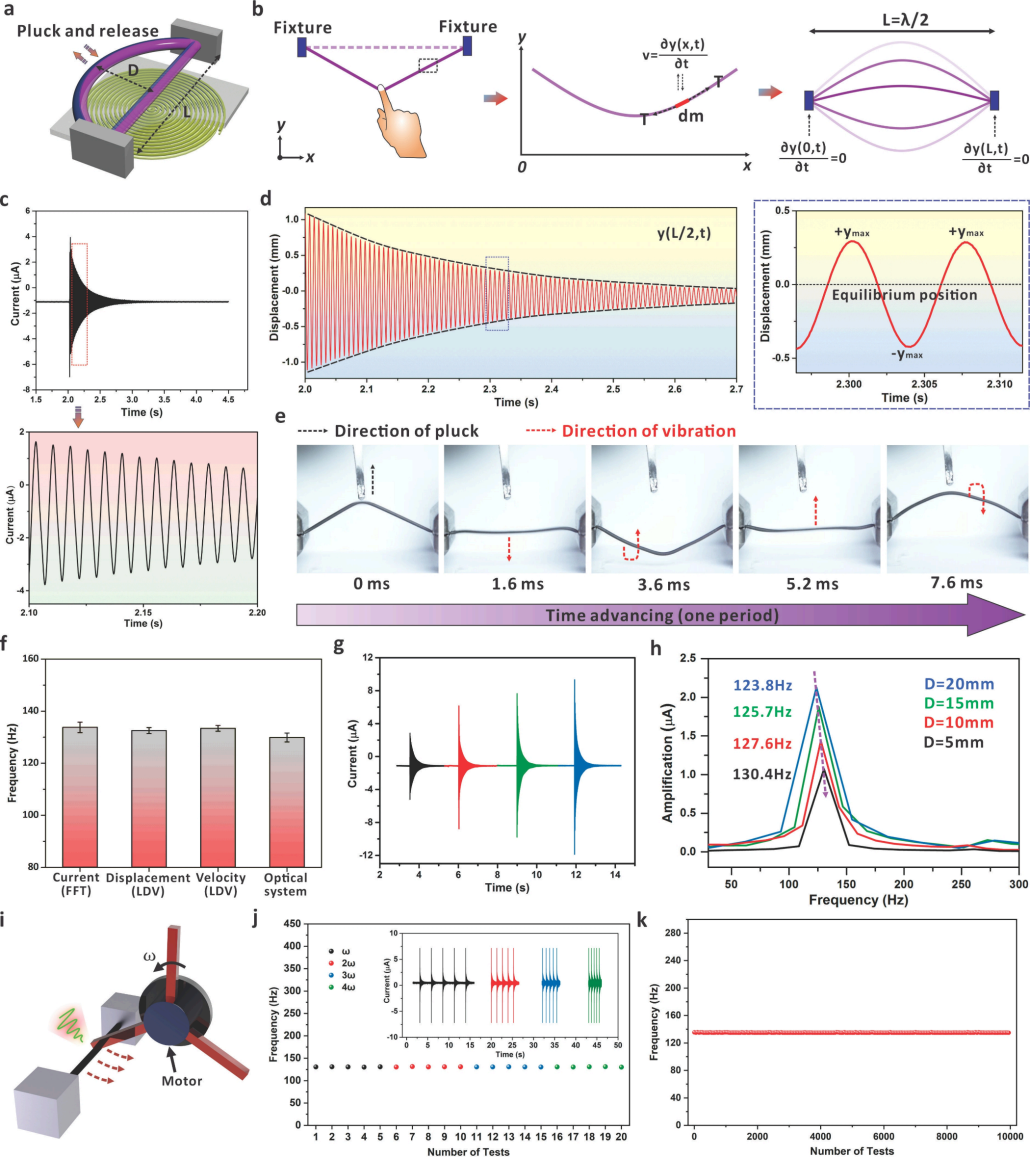
Characterization of eigenfrequency performance of magnetized strings. (a) Schematic diagram of the experimental setup for characterization of eigenfrequency performance. The string has length *L*, and it is plucked with a maximum lateral displacement of *D* to trigger the vibration. (b) Schematic diagram of the fundamental frequency of string vibration, which is the specific vibration frequency with only one wave valley as the largest amplitude. (c) Current signal generated in the coil when the magnetized string vibrates. (d) Behavior of displacement variation at the center point of the magnetized string recorded by an LDV. (e) Vibration process of the magnetized string recorded by a high-speed camera. (f) Eigenfrequency of the magnetized string measured by four different methods. (g) Four current signals generated by magnetized string vibration with different lateral stretching distances of *D*. (h) Frequency spectra corresponding to the current profiles in (g). (i) Schematic diagram of the experimental setup to periodically pluck the magnetized string for signal analysis. The plucking speed is controlled by the electrical motor. (j) Current signals and corresponding eigenfrequencies generated by the magnetized string at different plucking speeds. (k) Eigenfrequencies recorded during 10,000 vibrations of the magnetized string. Every 50 data points is plotted as a point.

To quantitatively characterize the mechanical properties of the string vibration and its relationship with the electric information, e.g., eigenfrequency, a magnetized string with length *L* was applied for the investigation (Figure 2a). The center of the magnetized string was manually plucked along the horizontal direction with a tweezer, and the string would vibrate accordingly once the mechanical constraint was released. As depicted in Figure 2b, we defined that the oscillation direction of the mass element (dm) on the string is along the *y*-axis, and the wave propagation is along the *x*-axis. The instantaneous oscillation velocity of the mass element on the magnetized string can then be defined as . Consequently, the two fixed ends of the string will always be static with zero instantaneous velocity . Note that when the two-end-fixed string is free to vibrate, standing waves of different frequencies are usually formed, where the fundamental frequency is the wave frequency with only one wave valley on the mechanical wave. In this work, the extracted eigenfrequency of the magnetized string refers to the fundamental frequency during the vibration. As a result, the length of the string can be considered equal to half the wavelength (λ/2) of the mechanical wave. Figure 2c shows a typical profile of current signals generated in the coil during string vibration. The magnetized string for study here had a typical mass ratio of PDMS, Ecoflex, and NdFeB particles (Mp:Me:Mn) at 2:3:5, and the radius of the string was set at 0.7 mm. The original length of the string was 30 mm, and it was stretched to 36 mm with tension to fix on top of the coil layer. As presented in the enlarged image, the electrical signals are in the form of a damped sinusoidal wave, which is tightly associated with the vibration of the magnetized string. The induced current signals can then be converted into the frequency domain by the FFT analysis method, and the frequency of the obtained signal is 130 Hz (Figure S5). Apart from the electrical signal, the vibration process of the magnetized string was further recorded by a laser Doppler vibrometer (LDV). Via focusing the laser spot on the central position of the string, Figure 2d shows the time–displacement relationship, which also exhibits the characteristics of a damped sinusoidal waveform. Once released, the string vibrates around the equilibrium position in the *y*-direction, resulting in corresponding magnetic flux variation to induce electrical signals in the coil underneath. With the FFT conversion, the eigenfrequency from the vibrating motion can be obtained, which exhibits the same peak position as the current signals (Figure S6). Meanwhile, the instantaneous velocities at the central position of the string exhibit consistent damping behavior and eigenfrequency during the vibration (Figure S7). In addition, the vibration behavior of the magnetized string throughout an entire period was recorded with a high-speed camera system (Video S1). As shown in Figure 2e, one vibration period of the magnetized string is ∼7.6 ms, which is consistent with the period of the electrical signal from the magnetized string. The consistency of eigenfrequencies from the above investigations proves the feasibility of obtaining the string eigenfrequency using the current signals by the principle of electromagnetic induction (Figure 2f).

The stability of the sensing response is critical to evaluate the reliability of the wearable HMI interface. In principle, eigenfrequency is an intrinsic property of the object and does not change with the excitation conditions. To test the stability of the eigenfrequency of the magnetized string, different excitation conditions were applied to the magnetized string. As depicted in Figure 2a, we stretched the magnetized string from the equilibrium position to different lateral displacements (*D*) and released it. Figure 2g presents the induced current signals, which exhibit a variation of amplitude that is positively related to the stretching distance (*D*). With a larger lateral displacement, the amplitude of electrical signals increases because of the more obvious variation of magnetic flux. However, the eigenfrequencies are located at similar peak positions after the FFT analysis (Figure 2h). Figure S8 further demonstrates the repeatability of the eigenfrequency when the magnetized string was plucked to different *D* values for five tests. Such a phenomenon confirms that eigenfrequency is an intrinsic property of the magnetized string and is not affected by the maximum displacement during manual operations. We also observed the small shift of the characteristic peak from the frequency spectra, which can be mainly attributed to air resistance during string vibration. With a larger stretching distance, the vibration speed becomes faster and more energy is consumed by air resistance, resulting in a slight decay of the vibration eigenfrequency.^33^ Since the magnetized string possesses a low elastic modulus, only a minimal force is required to trigger the vibration of the flexible string. We thus measured the force required to stretch the magnetized string from its equilibrium position to different distances. As shown in Figure S9, only ∼0.1 N is sufficient to stretch a typical magnetized string by 5 mm, which can enable a vibration process for eigenfrequency extraction after releasing. The results ensure that the effective vibration of the string for eigenfrequency extraction can be realized by manual operations. In real applications, users normally pluck the strings with a maximum displacement of several millimeters, and thus, the effect of the stretching distance on eigenfrequency variation can be ignored. Considering the difference in the user’s plucking position during daily use, five different positions on the magnetized string were plucked and the eigenfrequencies were recorded (Figure S10). The result shows that different plucking positions have almost no effect on the eigenfrequency. Similarly, the effect of different plucking angles on the eigenfrequency was further explored. The magnetized string exhibits the same eigenfrequency at plucking angles (θ) of 0°, 30°, 60°, and 90°, indicating that the plucking angle has no significant effect on the eigenfrequency (Figure S11). Here, θ is the angle between the plucking and the horizontal directions. In addition, the plucking speed by a human finger is another important parameter to evaluate the stability of the interface toward daily applications. As shown in Figure 2i, an experimental setup was designed to study the effect of plucking speed on the eigenfrequency, where the motor plucked the magnetized string periodically at an angular speed from ω to 4ω, and the eigenfrequencies were recorded by a LabVIEW script (Figure S12). Figure 2j shows the corresponding current signals produced by different angular speeds and the related eigenfrequencies obtained by FFT analysis. The results show that the current signals formed by the magnetized string vibration are significantly related to the plucking speed and faster plucking speeds lead to the faster generation of electrical signals. However, the obtained eigenfrequencies remain essentially constant, indicating that eigenfrequency is an intrinsic property and is unaffected by the plucking speed. This behavior is critical to ensure the reliability of the eigenfrequency-based mechanism because the plucking speed is difficult to maintain at a constant value during real applications. To test the robustness of signal generation, the magnetized string was plucked by an electrical motor ∼10,000 times, and the eigenfrequency was recorded simultaneously. As presented in Figure 2k, the eigenfrequency of the magnetized string maintains a stable value during the test, which exhibits the stability and accuracy of the magnetized string toward long-term usage for HMI. Furthermore, we tested the current signals generated by the same magnetized string while changing the coil layer to different sizes (1 mm × 1 mm, 2 mm × 2 mm, and 5 mm × 5 mm). The results in Figure S13 show that all measurements reveal a similar eigenfrequency of the vibrating string, confirming that the intrinsic string is critical for the eigenfrequency value instead of the coil dimension. Consequently, we could optimize the dimension of the coil layers according to different HMI applications in the future. This is important to further improve the flexibility of the entire assembly and thus the wearability experience in practical use. Considering the plucking habits of different users in daily use, four volunteers participated in the test by plucking the magnetized string for five individual tests (Figure S14). The recorded eigenfrequencies are essentially unchanged, indicating that the eigenfrequency-based mechanism has a reliable potential in the daily HMI toward different users.

### Regulation of Eigenfrequencies

**Figure 3:**
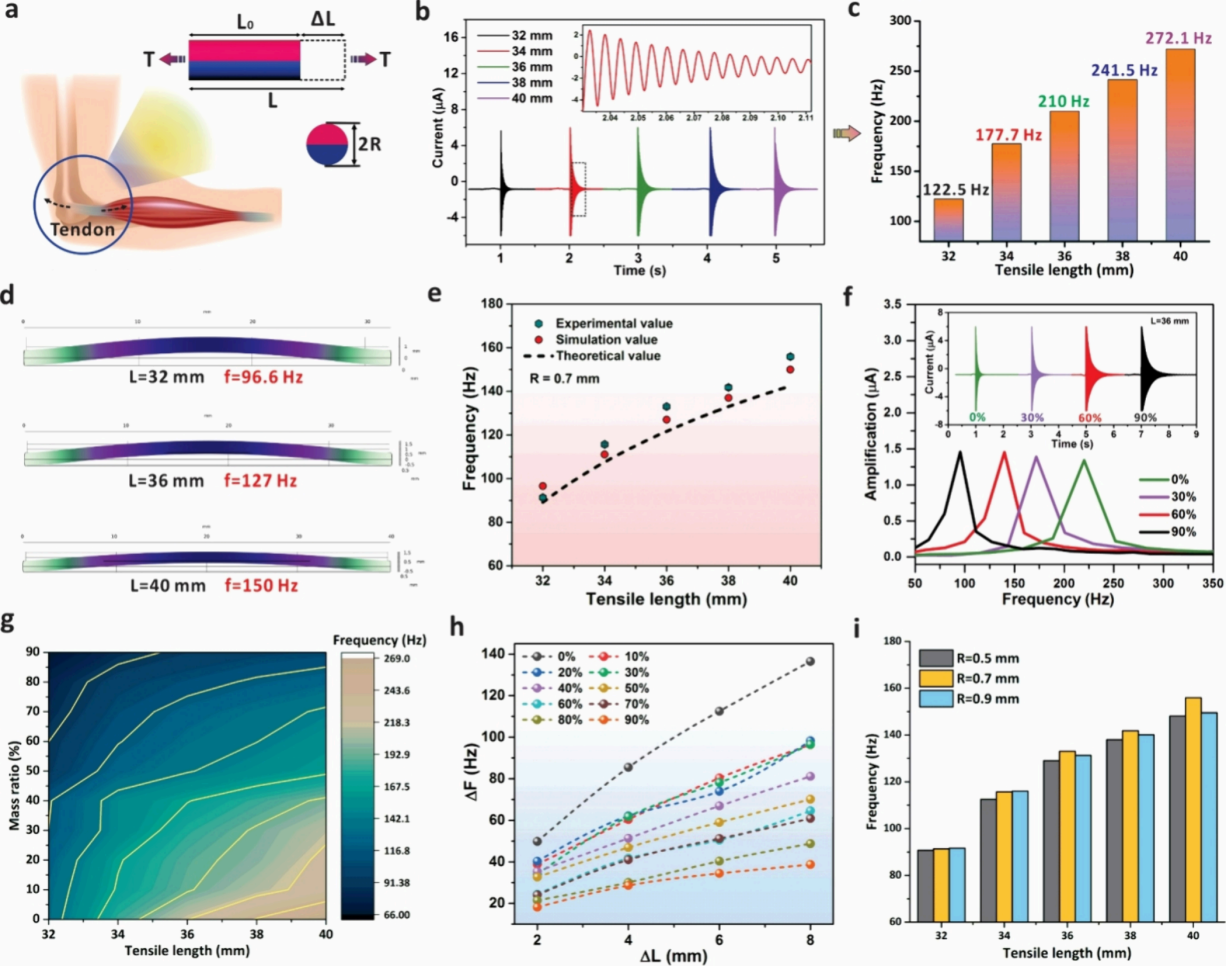
Regulation of magnetized string eigenfrequencies. (a) Schematic diagram of the adjustable parameters, tension, and tensile length from the magnetized string inspired by a tendon. (b) Induced current signals during vibration of the magnetized string at different tensile lengths. (c) Corresponding eigenfrequency values of the magnetized string under different tensile lengths. (d) Simulated eigenfrequencies of the magnetized string at different tensile lengths. (e) Comparison of the experimental value, simulation value, and theoretical value of eigenfrequency based on the same magnetized string. (f) Induced current signals during vibration of magnetized strings with different Ecoflex contents and the corresponding frequency spectra. (g) Eigenfrequencies of magnetized strings at different tensile lengths and different Ecoflex contents. (h) Effect of change in tensile length (Δ*L*) on eigenfrequency variation (Δ*F*). (i) Eigenfrequencies of magnetized strings with different radii under the same tensile lengths.

The regulation of eigenfrequency is essential for the assignment of commands in a reliable, multifunctional, and effective HMI system. As shown in Figure 3a, a string model is used to offer guidance on eigenfrequency regulations. Based on previous studies, the governing function for the eigenfrequency of a tensioned string can be described as^36^
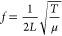
1where *L* is the tensile length and *T* and μ are the tension and the linear density of the string, respectively. For a string with a fixed initial length (*L*_0_), the change in tension (*T*) and linear density (μ) of the string is tightly related to the change in tensile length (*L* = *L*_0_ + Δ*L*, where Δ*L* is the relative change in length). Such a relationship between the tension and tensile length of a stretchable string is also well-known in the tendon system, as depicted in Figure 3a. Consequently, we first investigated the dependence of eigenfrequency on the tensile length (*L*). To study the length effect, a magnetized string with an initial length of 30 mm was stretched to different lengths, and the induced current signals during the vibration process were recorded. Figure 3b shows the current signals at different tensile lengths, which exhibit the characteristics of damped sinusoidal waveforms at different stretching lengths. Here, the magnetized string was prepared in the mass ratio of Mp:Me:Mn at 2:3:5 with a radius of 0.7 mm. The frequency spectra corresponding to the current signals are provided in Figure S15. The results show that the eigenfrequency of the magnetized string increases along with the increase in the tensile length (Figure 3c). When the string length was stretched from 32 to 40 mm, the eigenfrequency increased from ∼122.5 to ∼272.1 Hz. Thus, the tensile length can serve as an effective parameter for eigenfrequency control in various HMI scenarios. We further measured the tension of the magnetized string during stretching and applied the results to Equation 1 for theoretical eigenfrequency calculation (Figure S16, see Note S1 for the detailed derivation process). In addition, COMSOL Multiphysics was applied to simulate the eigenfrequency values of the magnetized string (Figures 3d and S17). When the tensile length varies from 32 to 40 mm, the eigenfrequency changes in a similar behavior as the experimental observation. The theoretical and simulation results are plotted in Figure 3e, which is consistent with the experimental values under different tensile lengths. The comparison also confirms that the vibration eigenfrequency of the magnetized string can be accurately predicted based on the model to ensure nonoverlapping frequency design for multifunctional HMI applications.

Apart from tensile length, elastic modulus (*E*) of the magnetized string is another important parameter that influences the eigenfrequency value. The elastic modulus of the magnetized string can be changed by precisely adjusting the mass ratio of the PDMS and Ecoflex in the composite. We herein fixed the mass of NdFeB particles equal to the sum of PDMS and Ecoflex while tuning the ratio of PDMS and Ecoflex contents for elastic modulus regulation (Figure S18). With a higher content of Ecoflex, the elastic modulus of the magnetized string decreases, resulting in a smaller eigenfrequency value, as presented in Figure 3f. For example, the magnetized string with an Ecoflex content of 0% has an eigenfrequency value of ∼220.1 Hz, while the string with the highest Ecoflex content of 90% exhibits an obvious eigenfrequency decrease to ∼95.2 Hz. The main cause of this phenomenon is attributed to the smaller tension that is required to stretch the magnetized strings with a low elastic modulus under the same tensile length (36 mm). Figure 3g summarizes the eigenfrequencies of magnetized strings with different Ecoflex contents (*E* control) and tensile lengths (*L* control). The results show that the eigenfrequency of the magnetized string has a broad adjustable range based on the above parameters, which can be effectively applied for the allocation of different commands in HMI systems. From Figure 3g, it also can be concluded that a larger string tension (tensile length) and larger elastic modulus (smaller mass ratio of Ecoflex) lead to the increase of the eigenfrequency. In one way, a higher eigenfrequency is related to a larger tensile length (tension) if the mass ratio of PDMS/Ecoflex is fixed. In another way, a higher eigenfrequency can also be obtained with a smaller content of Ecoflex (higher elastic modulus) if the string is stretched by the same tensile length. Figure 3h summarizes the variation of eigenfrequency (Δ*F*) for strings with different tensile lengths when the devices were prepared by different Ecoflex contents from 0 to 90%. The results indicate that with a larger elastic modulus of the magnetized string, the value of Δ*F* will be larger for the same Δ*L*. As will be discussed below, for HMI systems based on a single magnetized string, the accurate assignment of commands relies mainly on the tensile length and applied tension. Consequently, strings with a relatively higher elastic modulus can be chosen for a single-string-based system because they are strongly influenced by the tensile length and applied tension. Furthermore, the eigenfrequencies of strings with radii of 0.5 and 0.9 mm were also measured based on different Ecoflex contents and tensile lengths (Figure S19). The results show that compared with the tensile length and elastic modulus, the effect from the string radius can generally be ignored (Figure 3i). This can also be explained by the model of string vibration as discussed in Note S2, where the parameter of radius was finally eliminated from the governing function. As demonstrated in Figure S20, the theoretical values and simulation values of eigenfrequency also match well with the experimental values when the radius of the magnetized string was changed to 0.5 and 0.9 mm. Consequently, the magnetized string with an appropriate radius can be determined according to the spatial requirement to ensure the comfort of wearable experience for different HMI situations. The results experimentally confirm that the eigenfrequency of a specific magnetized string can be predicted based on the established model. Furthermore, the immunity to manual parameters, e.g., plucking amplitude, speed, etc., ensures that the eigenfrequency would not be significantly affected during the operations. This is essential for accurate HMI applications because it sets the stage to allocate related commands based on the eigenfrequency-dominant mechanism.

### HMI Demonstrations by the Eigenfrequency-Based String System

**Figure 4:**
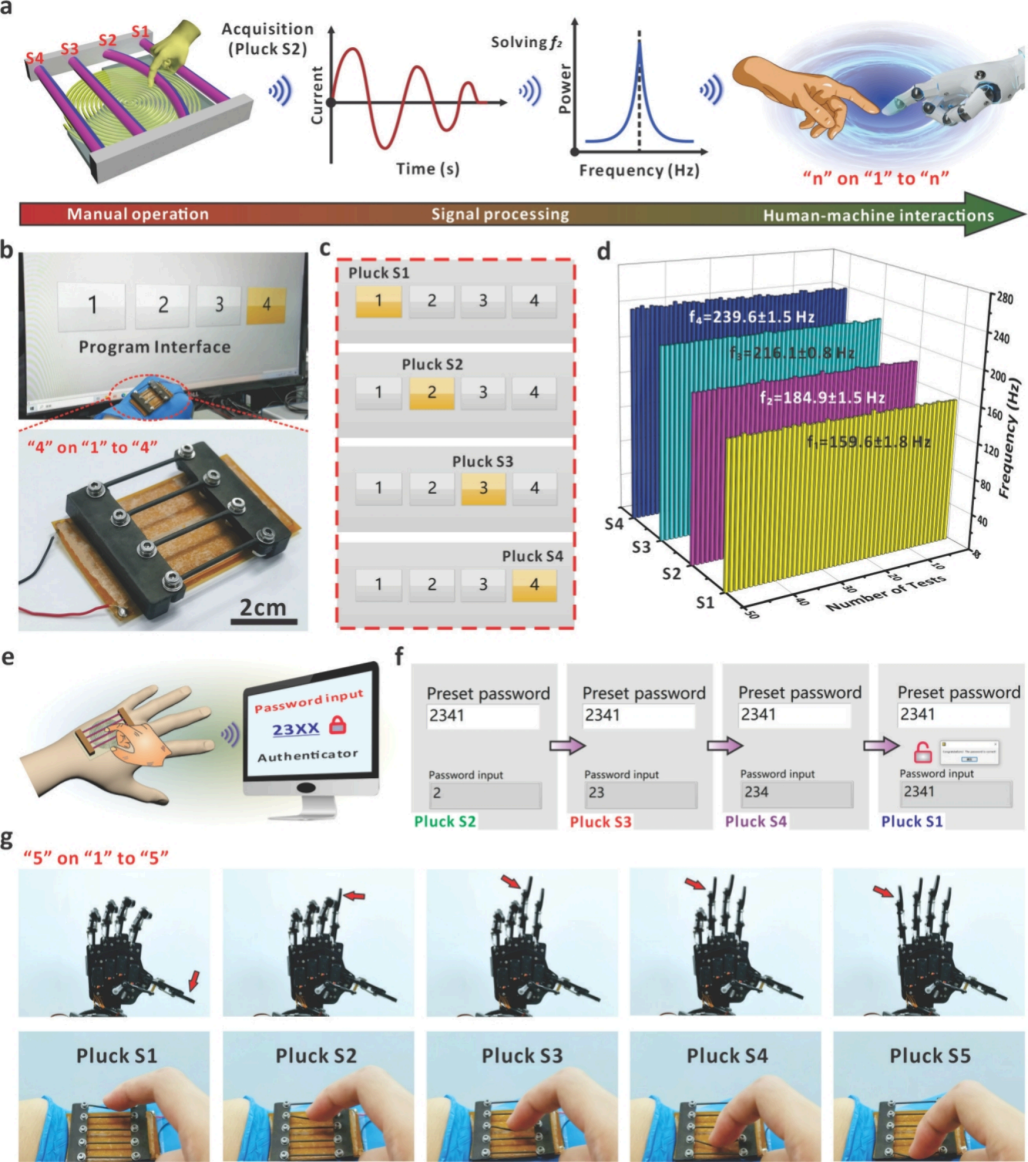
Demonstration of HMI systems based on multiple magnetized strings with customized eigenfrequencies. (a) Schematic diagram of the operation processes for HMI systems with four magnetized strings from the manual operation and signal processing to HMIs. (b) Optical image of the device assembled from four magnetized strings and the corresponding program interface for HMI. (c) Images of the program interface when different strings were plucked and the corresponding numbers were addressed. (d) Eigenfrequencies recorded by plucking four magnetized strings 50 times. The eigenfrequencies were recorded by a LabVIEW script to obtain the average value. (e) Schematic diagram of the interface for the password authentication system based on four magnetized strings. (f) Successful process of unlocking the system with the password “2341” using the interface based on four strings. (g) Process of controlling five robotic fingers by the HMI device with five magnetized strings. Each command of finger operation was encoded with one specific eigenfrequency.

As discussed above, the eigenfrequency of the magnetized string can be effectively regulated based on the property combination. With only one shared conductive coil, multiple commands could thus be transmitted to different terminals to realize specific functions. Figure 4a shows a schematic diagram of the wearable HMI interface composed of multiple magnetized strings (each with a customized eigenfrequency) with one coil layer underneath. When a specific string (e.g., S2) is plucked by a human finger, the current with the corresponding eigenfrequency would be induced within the coil. After signal acquisition and processing, the specific command can be sent to the electrical terminal to realize various interactive operations that has been encoded with the solved frequency. As a proof of concept, we assembled four magnetized strings on the coil and connected the interface with the LabVIEW interface. The “4” on “1” to “4” interface consists of four strings with different moduli and stretching lengths to realize identifiable eigenfrequencies for reliable command coding (Figure 4b). As shown in Figure 4c, when the magnetized string with a specific eigenfrequency was plucked, the interface received the electrical signals and analyzed the eigenfrequencies to highlight the corresponding numbers 1–4 (Video S2). Such addressing results confirmed that the eigenfrequency could be allocated with a specific function for reliable and convenient HMI applications. To demonstrate the stability of the wearable interface, each magnetized string was plucked 50 consecutive times, and the frequencies of the magnetized strings were recorded using the LabVIEW script. As presented in Figure 4d, the frequencies of the four specific strings are 159.6 ± 1.8, 184.9 ± 1.5, 216.1 ± 0.8, and 239.6 ± 1.5 Hz. This indicates that the four-string-based system has remarkable discrimination and stability for high information capacity HMI (“4” on “1” to “4”). In principle, more magnetized strings can be integrated into the interface to obtain a higher interactive capacity, which can be simply realized by customizing the property of the string array. Based on the above digit generation capability, a password authentication system was designed using a single device as the input (Video S3). Figure 4e shows the process of system password inputting and unlocking by using the different eigenfrequencies of the four magnetized strings. First, we defined “2341” as the preset password in the system and then plucked the strings of S2, S3, S4, and S1 in turn, which could generate the corresponding numbers of 2, 3, 4, and 1 in sequence for system input based on the received eigenfrequency (Figure 4f). When the input password matched the preset password, the system was unlocked successfully. Similarly, the setting of higher security passwords can be achieved by increasing the number of magnetized strings of different eigenfrequencies. As the design requires only one electrical output to transmit the signals for eigenfrequency determination, increasing the number of magnetized strings and digits will not bring unnecessary burdens to the interaction system. In addition, the design has high practicability for robot control (“5” on “1” to “5”). We designed an HMI device consisting of five magnetized strings, and by connecting the robotic hand to the LabVIEW interface, the extension of each finger can be precisely controlled. As shown in Figure 4g, when the magnetized strings of S1–S5 were continuously plucked, the successful transmission of five different eigenfrequencies could result in the extension of five robotic fingers in turn (Video S4). The demonstrations show that the eigenfrequency-based mechanism could enable an effective and functional interaction behavior thanks to the facile frequency customization of the oscillating strings. Note that during the manual operations it is possible that users might touch the neighboring string by mistake. Considering this, we performed the experiment by simultaneously plucking two strings to evaluate the accuracy of the system. As shown in Figure S21, two magnetized strings with eigenfrequencies of 150 and 230 Hz are plucked at the same time via different plucking strengths. After the FFT analysis, two eigenfrequencies can be extracted, as presented in the spectra. Because the designed program can only identify one eigenfrequency at one time, the frequency with a lower amplitude will not be recognized, and only the intensified eigenfrequency will be considered as the input from the user. This ensures that the correct command can be delivered to the terminal to trigger related operations. Therefore, if other magnetized strings are touched by mistake during the plucking operation, the smaller vibrations will not affect the delivery of the intended command that is related to the string under normal plucking action.

**Figure 5:**
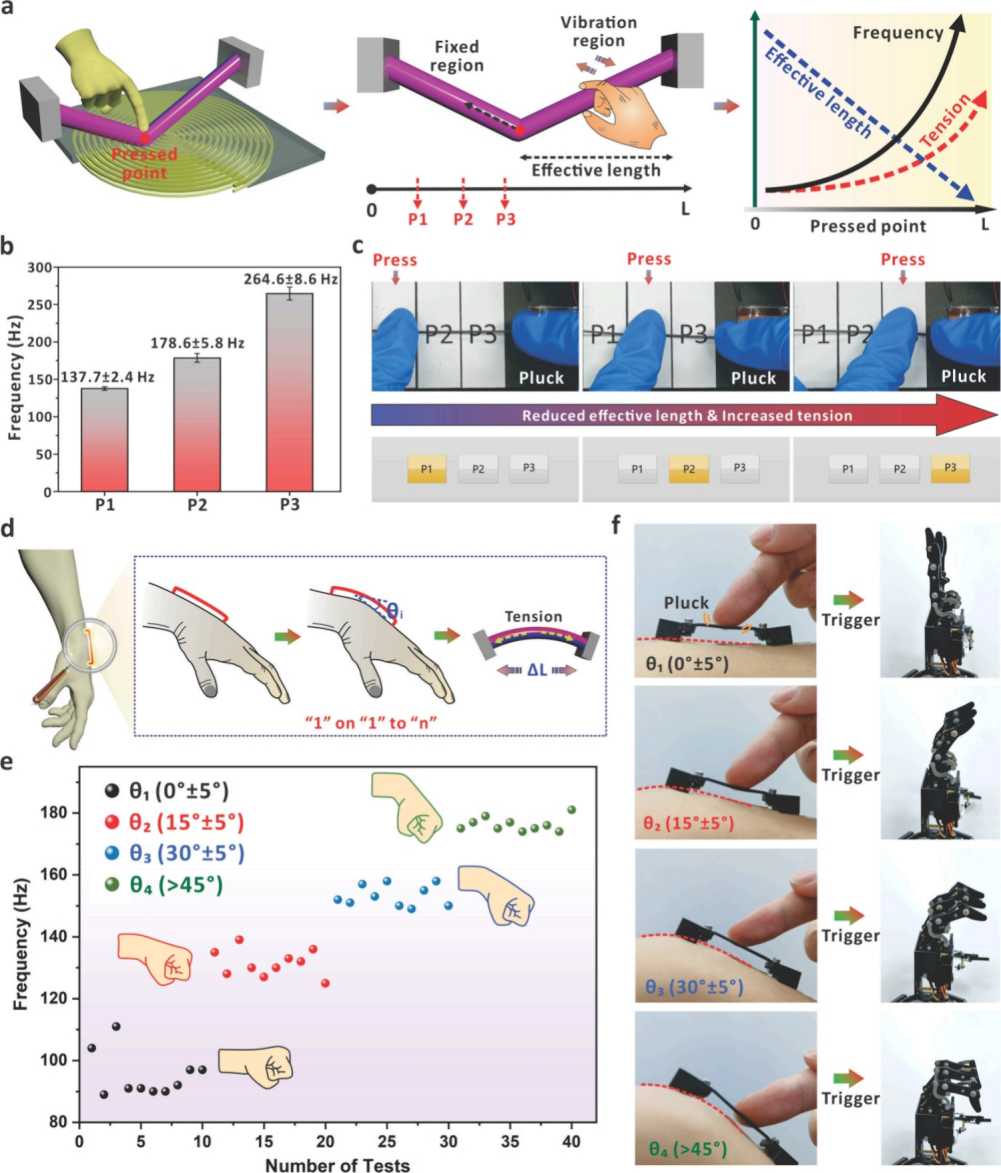
Demonstrations of tension-regulated HMI systems based on a single magnetized string. (a) Schematic diagram and principle of changing the eigenfrequency of a magnetized string by pressing different positions of the string. (b) Eigenfrequency of the magnetized string in the vibration region when it was pressed at three different positions. (c) Process of lighting up the corresponding addresses by pressing different positions with different eigenfrequency productions. (d) Schematic diagram of changing the eigenfrequency of a magnetized string by bending the human wrist to regulate the tension and length of the magnetized string. (e) Eigenfrequencies of the magnetized string under different applied tensions by changing the wrist bending angles. (f) Record of controlling the bending of robotic fingers based on different stretching status of the string via wrist bending.

It has been proven that magnetized strings have excellent tensile properties, and there is a significant relationship between the eigenfrequency and the tensile length. These attributes can be introduced to further relieve the hardware configuration and broaden the operation modes of HMI devices based on magnetized strings. Figure 5a shows one approach that can be used to change the eigenfrequency of an oscillating magnetized string by applying a constraint on different positions. When a finger presses the magnetized string at a specific location, the string is divided into two regions including the fixed and the vibration region. As the pressing point moves to the right end, the tension on the string in the vibration region increases with the decreased effective length, and the eigenfrequency increases significantly according to the string vibration model. As a proof of concept, a magnetized string with an original length of 35 mm and 90% Ecoflex content was stretched to 100 mm and fixed at both ends. Three positions, P1, P2, and P3, were marked on the bottom plane, and the vertical separation between the string and the bottom plane was 8 mm. When pressing the string at a specific point, the finger with the pressed point was confirmed to touch the bottom surface so that the repeatability of string tension in the vibrating region can be ensured. To test the reliability of the interface, the magnetized string was vibrated 10 times at each pressed position, and the LabVIEW script was used to record the string frequency (Figure 5b). When P1, P2, and P3 were pressed, the recorded eigenfrequencies were 137.7 ± 2.4, 178.6 ± 5.8, and 264.8 ± 8.6 Hz, indicating the customization potential of eigenfrequencies for command assignment in HIM systems. As presented in Figure 5c, when the positions of P1, P2, and P3 were pressed and the string in the vibration region was plucked, respectively, the corresponding area in the LabVIEW interface would be highlighted (Video S5). In this process, only one magnetized string was needed for the output of different frequencies and commands. Consequently, the assignment of commands in HMI systems based on a single magnetized string can be achieved intuitively using the flexibility of human joints. As shown in Figure 5d, for a magnetized string fixed on the wrist, the length and tension of the magnetized string can be changed with the bending angle of the wrist. The eigenfrequency can thus be altered correspondingly to realize the “1” on the “1” to “*n*” HMI interface. Note that in this interaction mode, the assignment of commands depends on the tunable eigenfrequencies that can be regulated via different tensions and tensile lengths. As discussed above, the variation of eigenfrequency from the string with a large modulus is more obvious if the string has been stretched by the same amount in length (Figure 3h). Consequently, we chose the magnetized string with a larger elastic modulus to ensure distinguishable signal production during the application of tensions. We herein fixed the magnetized string (original length 30 mm and 20% Ecoflex content) at the human wrist and measured the eigenfrequency of the string under different wrist bending angles. Figure 5e shows that the eigenfrequencies of the string exhibited stable and repeatable values with corresponding wrist bending angles at 0° ± 5°, 15° ± 5°, 30° ± 5°, and >45°. It matches well with the theoretical model that a larger extended length and tension would lead to a higher eigenfrequency during the vibration process. Based on the above properties, manipulation of the robot can be simply achieved via one device with bending control of the human wrist. When the wrist was bent to different angles, the tensile length of the deposited string and the related tension would change accordingly. This finally resulted in the eigenfrequency change when the string was manually vibrated, and the emitted frequency signal could thus be applied as specific trigger for command allocation. As demonstrated in Figure 5f, by plucking the magnetized string on the wrist with different bending degrees, the bending behavior of the robotic fingers can be accurately and graphically mapped (Video S6). Compared with previous reports on wearable HMI interfaces,^37,38^ the tension-based approach requires only one sensing unit and one communication channel to output multiple and identifiable commands, which significantly simplifies the whole communication system toward a more effective and intuitive future. We also notice that the screw-based fixture might bring about the concern of wearable comfort in specific applications. For future optimizations, the fixture can also be possibly replaced by another flexible adhesive to ensure the overall flexibility of the assembly and enhance the wearing experience during HMI applications.

## Conclusions

In summary, inspired by the structure of a natural tendon, we designed a wearable HMI interface using the eigenfrequency of magnetized strings as sensing fundamentals. Coupled with electromagnetic induction and the coil layer, the vibration of a magnetized string can induce periodic current signals that allow the extraction of inherent eigenfrequency through a standard time–frequency signal conversion. Using the string vibration model, the eigenfrequencies of two-end-fixed magnetized strings can be effectively controlled by designing the elastic modulus, tensile length, tension, etc. The identifiable eigenfrequency of an individual string can then be allocated with a specific command for HMI applications with enhanced storage capacity. By the assembly of magnetized strings with different eigenfrequencies, multiple commands were successfully produced and transmitted through one signal channel. The demonstrations confirm that the design has potential applications in password authentication and robot control, and increasing the number of magnetized strings would not bring an exceedable burden on the system. In addition, we demonstrated the dependence of eigenfrequency on string tension and presented the strategies for outputting multiple commands through a single magnetized string due to the excellent stretching performance and frequency controllability. This behavior effectively simplifies the configuration of the interaction system, from connecting electrodes to communication channels. Along with the proven reliability and robustness, we anticipate that the methodology will promote the development of flexible devices in future HMI, especially where simple structures with high storage capacity are urgently required.

## Data Availability

Data supporting the findings of this study are available within the article (and its Supporting Information) and from the corresponding authors upon reasonable request.

## Supporting Information Available

Schematic diagram of the fabrication process of magnetized strings; SEM image and EDS of magnetized strings; changes in the magnetic field intensity around a magnetized string when plucked; optical image of the flexible coil electrode; frequency spectrum of the induced current signal and the time–displacement curve; velocity variation at the center point of the magnetized string and the corresponding frequency spectra; eigenfrequencies of the magnetized string at different stretching distances; force required to stretch the magnetized string from its equilibrium position to different distances; eigenfrequencies of the magnetized string at different plucking positions and under different plucking angles; LabVIEW script interface; induced currents under different sizes of coils and corresponding frequency spectra; eigenfrequencies of the magnetized string when manually plucked by four volunteers; frequency spectra corresponding to the induced current signals of the magnetized string at different tensile lengths; recorded tension during the stretching of a magnetized string; simulated eigenfrequencies of the magnetized string at tensile lengths of 34 mm and 38 mm; elastic modulus of magnetized strings with different Ecoflex contents; eigenfrequencies of magnetized strings with radii of 0.5 mm and 0.9 mm at different tensile lengths and Ecoflex contents; comparison of the experimental values, simulation values, and theoretical values of eigenfrequencies with radii of 0.5 mm and 0.9 mm; current signals and corresponding frequency spectra when two magnetized strings are plucked simultaneously (PDF)
Vibration process of a magnetized string (MP4)
Process of outputting four commands using four magnetized strings (MP4)
Password input and unlocking process using four magnetized strings (MP4)
Robotic hand controlled by five magnetized strings (MP4)
Sensing process of the pressed position on a single magnetized string (MP4)
Robotic hand controlled by bending the wrist (MP4)

## References

[ref1] Guo H.; Wan J.; Wang H.; Wu H.; Xu C.; Miao L.; Han M.; Zhang H. Self-Powered Intelligent Human-Machine Interaction for Handwriting Recognition. Research 2021, 2021, 4689869 10.34133/2021/4689869. 33880448 PMC8035911

[ref2] Heng W.; Solomon S.; Gao W. Flexible Electronics and Devices as Human–Machine Interfaces for Medical Robotics. Adv. Mater. 2022, 34, e2107902 10.1002/adma.202107902. 34897836 PMC9035141

[ref3] Lin Z.; Zhang G.; Xiao X.; Au C.; Zhou Y.; Sun C.; Zhou Z.; Yan R.; Fan E.; Si S.; Weng L.; Mathur S.; Yang J.; Chen J. A Personalized Acoustic Interface for Wearable Human–Machine Interaction. Adv. Funct. Mater. 2022, 32, 2109430 10.1002/adfm.202109430.

[ref4] Shi Q.; Zhang Z.; Yang Y.; Shan X.; Salam B.; Lee C. Artificial Intelligence of Things (AIoT) Enabled Floor Monitoring System for Smart Home Applications. ACS Nano 2021, 15, 18312–18326. 10.1021/acsnano.1c07579. 34723468

[ref5] Zengeler N.; Kopinski T.; Handmann U. Hand Gesture Recognition in Automotive Human–Machine Interaction Using Depth Cameras. Sensors 2019, 19, 59 10.3390/s19010059. PMC6339101 30586882

[ref6] Zhao X.; Askari H.; Chen J. Nanogenerators for smart cities in the era of 5G and Internet of Things. Joule 2021, 5, 1391–1431. 10.1016/j.joule.2021.03.013.

[ref7] Zhou H.; Huang W.; Xiao Z.; Zhang S.; Li W.; Hu J.; Feng T.; Wu J.; Zhu P.; Mao Y. Deep-Learning-Assisted Noncontact Gesture-Recognition System for Touchless Human-Machine Interfaces. Adv. Funct. Mater. 2022, 32, 2208271 10.1002/adfm.202208271.

[ref8] Zhu M.; Sun Z.; Zhang Z.; Shi Q.; He T.; Liu H.; Chen T.; Lee C. Haptic-feedback smart glove as a creative human-machine interface (HMI) for virtual/augmented reality applications. Sci. Adv. 2020, 6, eaaz8693 10.1126/sciadv.aaz8693. 32494718 PMC7209995

[ref9] Xu C.; Chen J.; Zhu Z.; Liu M.; Lan R.; Chen X.; Tang W.; Zhang Y.; Li H. Flexible Pressure Sensors in Human–Machine Interface Applications. Small 2024, 20, 2306655 10.1002/smll.202306655. 38009791

[ref10] Ji B.; Mao Y.; Zhou Q.; Zhou J.; Chen G.; Gao Y.; Tian Y.; Wen W.; Zhou B. Facile Preparation of Hybrid Structure Based on Mesodome and Micropillar Arrays as Flexible Electronic Skin with Tunable Sensitivity and Detection Range. ACS Appl. Mater. Interfaces 2019, 11, 28060–28071. 10.1021/acsami.9b08419. 31306581

[ref11] Zhou Q.; Ji B.; Wei Y.; Hu B.; Gao Y.; Xu Q.; Zhou J.; Zhou B. A bio-inspired cilia array as the dielectric layer for flexible capacitive pressure sensors with high sensitivity and a broad detection range. J. Mater. Chem. A 2019, 7, 27334–27346. 10.1039/C9TA10489E.

[ref12] Lei M.; Ji B.; Ding S.; Liu R.; Zhou B. Template-free Formation of Hybrid Dielectric for Flexible Capacitive Sensors with Wide-Pressure-Range Linear Detection. Adv. Mater. Technol. 2023, 8, 2301077 10.1002/admt.202301077.

[ref13] Zhao D.; Zhang K.; Meng Y.; Li Z.; Pi Y.; Shi Y.; You J.; Wang R.; Dai Z.; Zhou B.; Zhong J. Untethered triboelectric patch for wearable smart sensing and energy harvesting. Nano Energy 2022, 100, 107500 10.1016/j.nanoen.2022.107500.

[ref14] Zhou Y.; Qiu X.; Wan Z. a.; Long Z.; Poddar S.; Zhang Q.; Ding Y.; Chan C. L. J.; Zhang D.; Zhou K.; Lin Y.; Fan Z. Halide-exchanged perovskite photodetectors for wearable visible-blind ultraviolet monitoring. Nano Energy 2022, 100, 107516 10.1016/j.nanoen.2022.107516.

[ref15] Yan Y.; Hu Z.; Yang Z.; Yuan W.; Song C.; Pan J.; Shen Y. Soft magnetic skin for super-resolution tactile sensing with force self-decoupling. Sci. Robot. 2021, 6, eabc8801 10.1126/scirobotics.abc8801. 34043530

[ref16] Ge J.; Wang X.; Drack M.; Volkov O.; Liang M.; Bermúdez G. S. C.; Illing R.; Wang C.; Zhou S.; Fassbender J.; Kaltenbrunner M.; Makarov D. A bimodal soft electronic skin for tactile and touchless interaction in real time. Nat. Commun. 2019, 10, 4405 10.1038/s41467-019-12303-5. 31562319 PMC6764954

[ref17] Yin R.; Wang D.; Zhao S.; Lou Z.; Shen G. Wearable Sensors-Enabled Human–Machine Interaction Systems: From Design to Application. Adv. Funct. Mater. 2021, 31, 2008936 10.1002/adfm.202008936.

[ref18] Shi Q.; Dong B.; He T.; Sun Z.; Zhu J.; Zhang Z.; Lee C. Progress in wearable electronics/photonics—Moving toward the era of artificial intelligence and internet of things. InfoMat 2020, 2, 1131–1162. 10.1002/inf2.12122.

[ref19] Kim J.; Kim S.; Park Y.-L. Single-input single-output multi-touch soft sensor systems using band-pass filters. npj Flexible Electron. 2022, 6, 65 10.1038/s41528-022-00201-8.

[ref20] Ren X.; Pei K.; Peng B.; Zhang Z.; Wang Z.; Wang X.; Chan P. K. L. A Low-Operating-Power and Flexible Active-Matrix Organic-Transistor Temperature-Sensor Array. Adv. Mater. 2016, 28, 4832–4838. 10.1002/adma.201600040. 27111745

[ref21] Song J.-K.; Son D.; Kim J.; Yoo Y. J.; Lee G. J.; Wang L.; Choi M. K.; Yang J.; Lee M.; Do K.; Koo J. H.; Lu N.; Kim J. H.; Hyeon T.; Song Y. M.; Kim D.-H. Wearable Force Touch Sensor Array Using a Flexible and Transparent Electrode. Adv. Funct. Mater. 2017, 27, 1605286 10.1002/adfm.201605286.

[ref22] Zhou Q.; Ji B.; Hu F.; Dai Z.; Ding S.; Yang H.; Zhong J.; Qiao Y.; Zhou J.; Luo J.; Zhou B. Magnetized Microcilia Array-Based Self-Powered Electronic Skin for Micro-Scaled 3D Morphology Recognition and High-capacity Communication. Adv. Funct. Mater. 2022, 32, 2208120 10.1002/adfm.202208120.

[ref23] Fang D.; Ding S.; Dai Z.; Zhong J.; Zhou B. Wearable patch with direction-aware sensitivity of in-plane force for self-powered and single communication channel based human-machine interaction. Chem. Eng. J. 2023, 468, 143664 10.1016/j.cej.2023.143664.

[ref24] Dai Z.; Feng K.; Wang M.; Lei M.; Ding S.; Luo J.; Xu Q.; Zhou B. Optimization of bidirectional bending sensor as flexible ternary terminal for high-capacity human-machine interaction. Nano Energy 2022, 97, 107173 10.1016/j.nanoen.2022.107173.

[ref25] Kim T.; Kim J.; You I.; Oh J.; Kim S.-P.; Jeong U. Dynamic tactility by position-encoded spike spectrum. Sci. Robot. 2022, 7, eabl5761 10.1126/scirobotics.abl5761. 35171645

[ref26] Guo H.; Wang H.; Xiang Z.; Wu H.; Wan J.; Xu C.; Chen H.; Han M.; Zhang H. Soft Human–Machine Interface with Triboelectric Patterns and Archimedes Spiral Electrodes for Enhanced Motion Detection. Adv. Funct. Mater. 2021, 31, 2103075 10.1002/adfm.202103075.

[ref27] Wei C.; Lin W.; Liang S.; Chen M.; Zheng Y.; Liao X.; Chen Z. An All-In-One Multifunctional Touch Sensor with Carbon-Based Gradient Resistance Elements. Nano-Micro Lett. 2022, 14, 131 10.1007/s40820-022-00875-9. PMC9198138 35699779

[ref28] Cao Y.; Yang Y.; Qu X.; Shi B.; Xu L.; Xue J.; Wang C.; Bai Y.; Gai Y.; Luo D.; Li Z. A Self-Powered Triboelectric Hybrid Coder for Human–Machine Interaction. Small Methods 2022, 6, 2101529 10.1002/smtd.202101529. 35084114

[ref29] Lei M.; Feng K.; Ding S.; Wang M.; Dai Z.; Liu R.; Gao Y.; Zhou Y.; Xu Q.; Zhou B. Breathable and Waterproof Electronic Skin with Three-Dimensional Architecture for Pressure and Strain Sensing in Nonoverlapping Mode. ACS Nano 2022, 16, 12620–12634. 10.1021/acsnano.2c04188. 35856940

[ref30] Sorokin S. V.; Grishina S. V.; Ershova O. A. Analysis and control of vibrations of honeycomb plates by parametric stiffness modulations. Smart Mater. Struct. 2001, 10, 1031 10.1088/0964-1726/10/5/320.

[ref31] Singh R. K.; Lye S. W.; Miao J. PVDF Nanofiber Sensor for Vibration Measurement in a String. Sensors 2019, 19, 3739 10.3390/s19173739. 31470572 PMC6749527

[ref32] Ding S.; Zhao D.; Chen Y.; Dai Z.; Zhao Q.; Gao Y.; Zhong J.; Luo J.; Zhou B. Single Channel Based Interference-Free and Self-Powered Human–Machine Interactive Interface Using Eigenfrequency-Dominant Mechanism. Adv. Sci. 2024, 11, 2302782 10.1002/advs.202302782. PMC10987133 38287891

[ref33] Ding S.; Fang D.; Liang Y.; Dai W.; Qi B.; Zhou B. Design of parallel coil arrays with identifiable eigenfrequency elements for wearable human-machine interactions. Appl. Mater. Today 2024, 36, 102039 10.1016/j.apmt.2023.102039. PMC11647967 39586297

[ref34] Zhang X.; Ai J.; Ma Z.; Du Z.; Chen D.; Zou R.; Su B. Magnetoelectric soft composites with a self-powered tactile sensing capacity. Nano Energy 2020, 69, 104391 10.1016/j.nanoen.2019.104391.

[ref35] Zhao X.; Chen G.; Zhou Y.; Nashalian A.; Xu J.; Tat T.; Song Y.; Libanori A.; Xu S.; Li S.; Chen J. Giant Magnetoelastic Effect Enabled Stretchable Sensor for Self-Powered Biomonitoring. ACS Nano 2022, 16, 6013–6022. 10.1021/acsnano.1c11350. 35417654

[ref36] Perov P.; Johnson W.; Perova-Mello N. The physics of guitar string vibrations. Am. J. Phys. 2016, 84, 38–43. 10.1119/1.4935088.

[ref37] Liu M.; Pu X.; Jiang C.; Liu T.; Huang X.; Chen L.; Du C.; Sun J.; Hu W.; Wang Z. L. Large-Area All-Textile Pressure Sensors for Monitoring Human Motion and Physiological Signals. Adv. Mater. 2017, 29, 1703700 10.1002/adma.201703700. 28949422

[ref38] Zheng K.; Gu F.; Wei H.; Zhang L.; Chen X. a.; Jin H.; Pan S.; Chen Y.; Wang S. Flexible, Permeable, and Recyclable Liquid-Metal-Based Transient Circuit Enables Contact/Noncontact Sensing for Wearable Human–Machine Interaction. Small Methods 2023, 7, 2201534 10.1002/smtd.202201534. 36813751

