# Supplementary material for: Bioinspired Magnetized String with Tension-Dependent Eigenfrequencies for Wearable Human–Machine Interactions
Source: ACS Appl Mater Interfaces. 2024 Nov 25;16(49):68465–77. doi: 10.1021/acsami.4c16653 (PMC11647967; doi:10.1021/acsami.4c16653)
Supplement: Supplementary file 1 — am4c16653_si_001.pdf [file am4c16653_si_001.pdf]

Supplementary Information

Bio-inspired Magnetized String with Tension-dependent Eigenfrequencies for Wearable Human-machine Interactions

Biao Qi,[1] Sen Ding,[1,*] Yuanzhe Liang,[1] Dan Fang,[1] Ming Lei,[1] Wenxue Dai,[1] Chao Peng,[2] Bingpu Zhou[1,3,*]

[1]Joint Key Laboratory of the Ministry of Education, Institute of Applied Physics and Materials Engineering, University of Macau, Avenida da Universidade, Taipa, Macau 999078, China

[2]School of Environmental and Chemical Engineering, Jiangmen Key Laboratory of Synthetic Chemistry and Cleaner Production and Institute of Carbon Peaking and Carbon Neutralization, Wuyi University, Jiangmen 529020, P.R. China

[3]Department of Physics and Chemistry, Faculty of Science and Technology, University of Macau, Avenida da Universidade, Taipa, Macau 999078, China

***Corresponding Author.**

Sen Ding, Email: yc07824@connect.um.edu.mo.

Bingpu Zhou, Email: bpzhou@um.edu.mo. Fax: +853-88222426. Tel: +853-88224196.

Contents

Supplementary Figures. 2

Supplementary Note 1. Theoretical eigenfrequency of the magnetized string. 23

Supplementary Note 2. Effect of radii on string eigenfrequencyies. 24

Supplementary Videos. 25

References 26

Supplementary Figures.

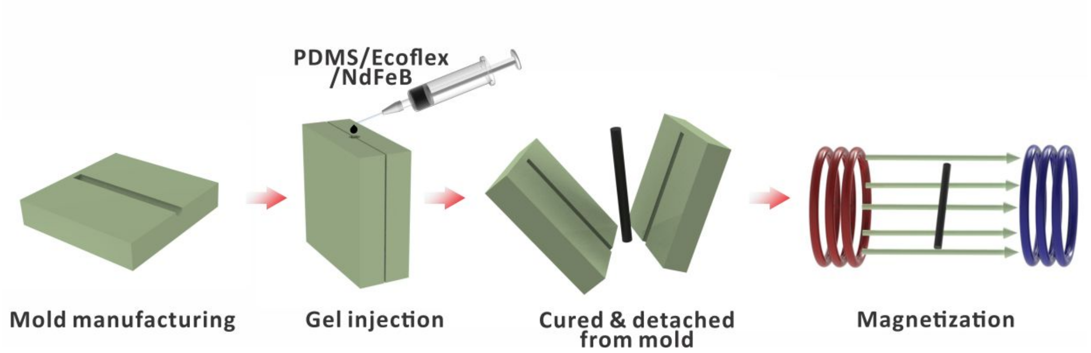

Figure S1. Schematic diagram of the fabrication process of magnetized strings. To enable a circular shape of the magnetized string, a semi-circular concavity was prepared on the plastic mold. By integrating two identical molds face-to-face, a hollow circular cylinder was obtained as shown in the figure. The injection and peeling-off of the composite can finally realize the cylindrical shape of the string for further magnetization. After magnetization, the string serves as a flexible and stretchable permanent magnet for mechanical to electrical conversion.

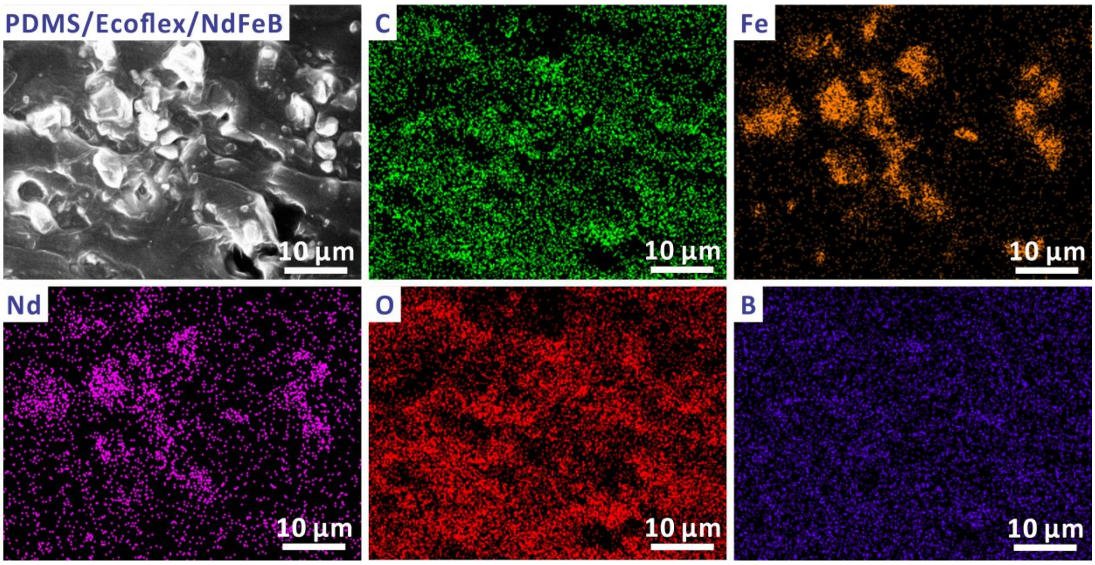

Figure S2. The Scanning Electron Microscope (SEM) image and Energy Dispersive Spectrum (EDS) of magnetized strings. The EDS results demonstrate the uniform distribution of NdFeB particles in the composite matrix.

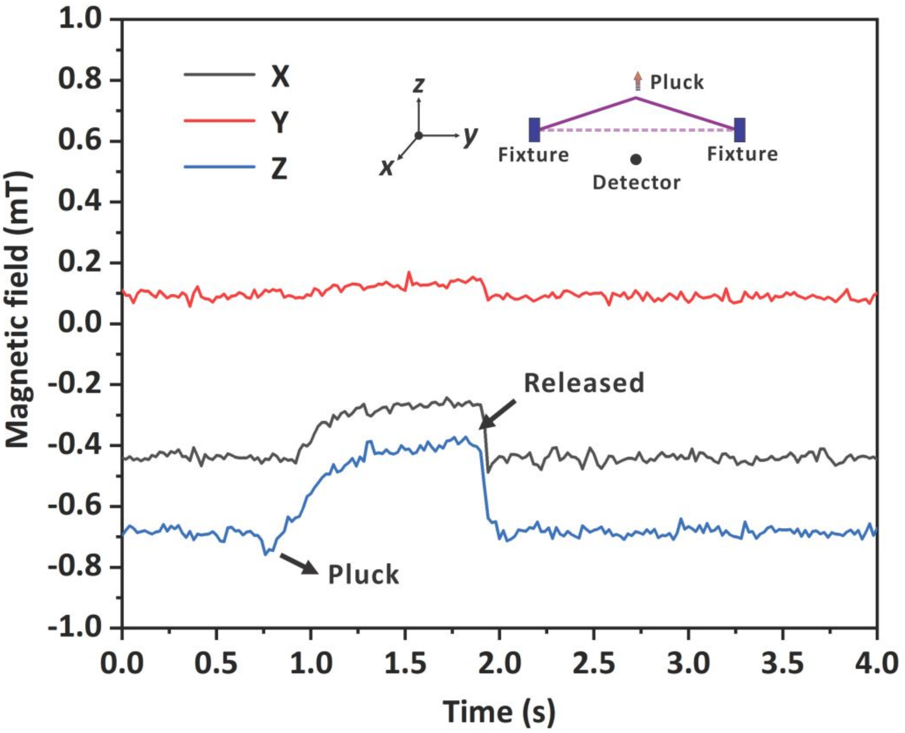

Figure S3. Changes in the magnetic field intensity around a magnetized string when it is manually plucked. The intensities in X, Y, and Z axes were measured in real-time via a commercial Gauss meter (the detector in the schematic diagram). The diagram of coordinate system is consistent with the X, Y, and Z axes in the plots.

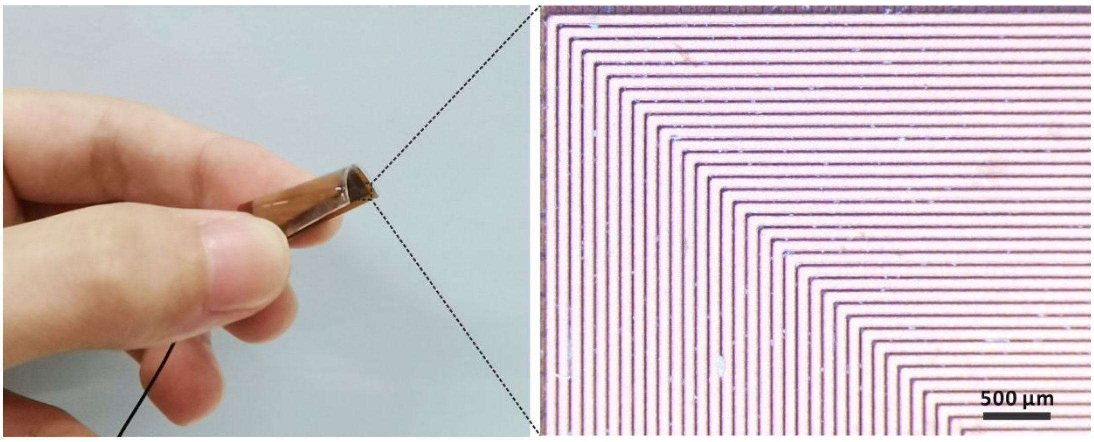

Figure S4. Optical image of the flexible coil electrode.

The width of each loop was 80 µm and the distance between two adjacent conductive lines was 20 µm. The coil is flexible and can be easily bent with recover capability.

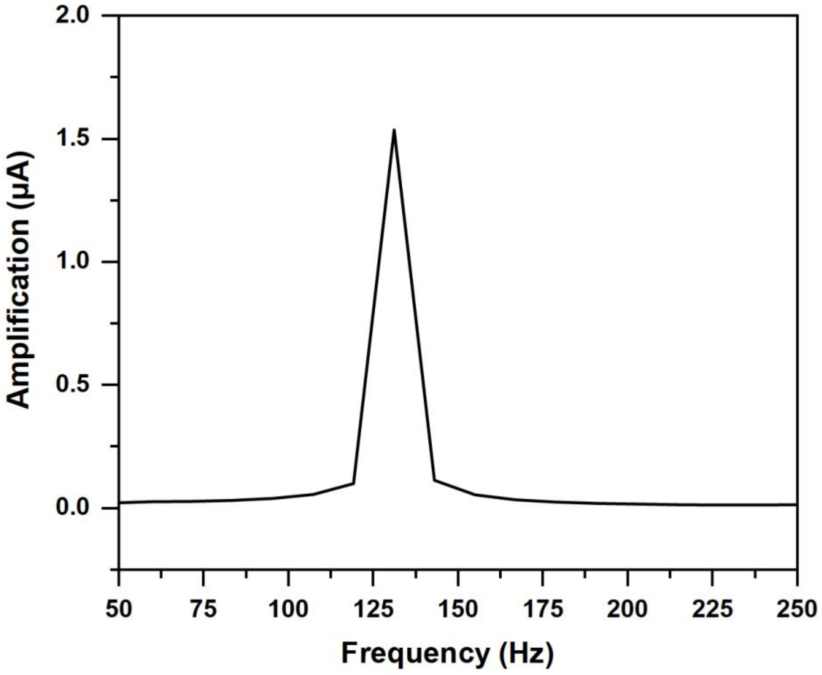

Figure S5. The frequency spectrum of the induced current signal by built-in FFT analysis method in OriginLab software.

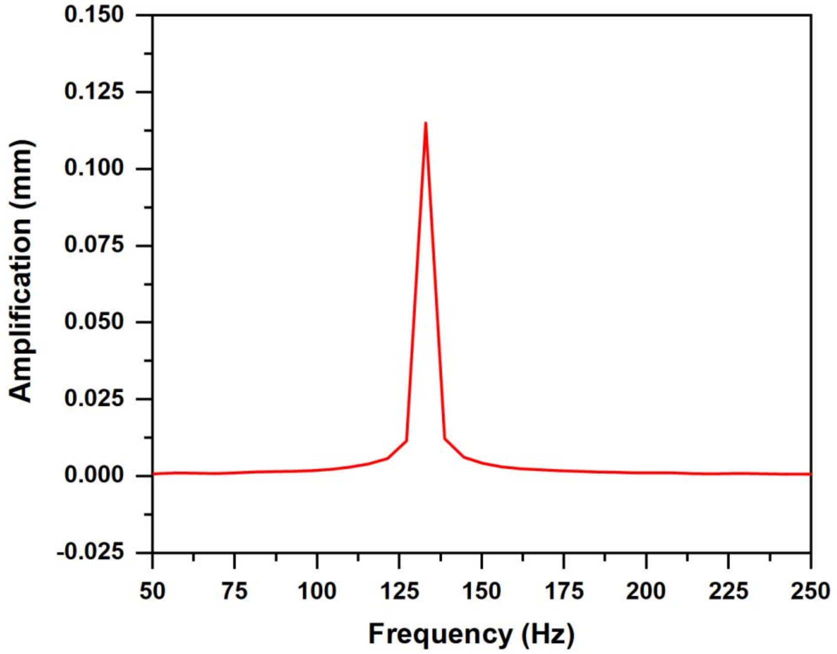

Figure S6. The frequency spectrum of the time-displacement curve by built-in FFT analysis method in OriginLab software. Related eigenfrequency is the same as the signal extracted from the electrical spectrum.

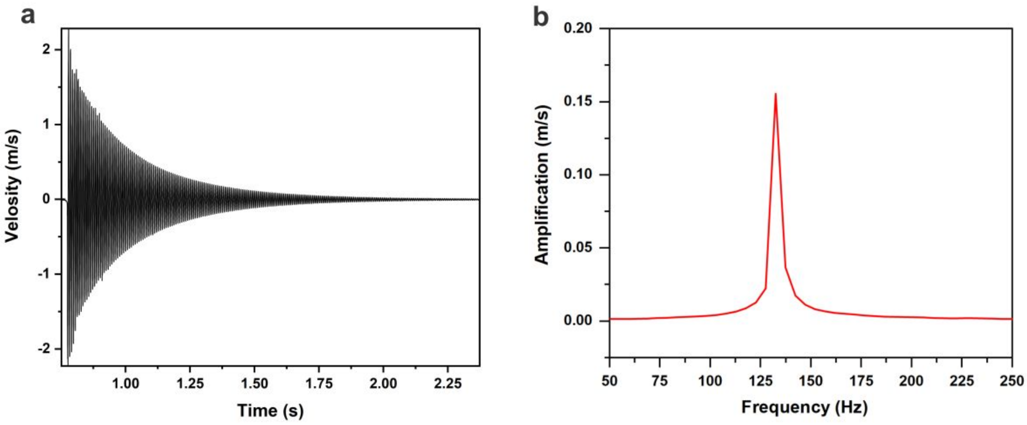

Figure S7. (a) Velocity variation at the center point of the magnetized string recorded by LDV and (b) the corresponding frequency spectra.

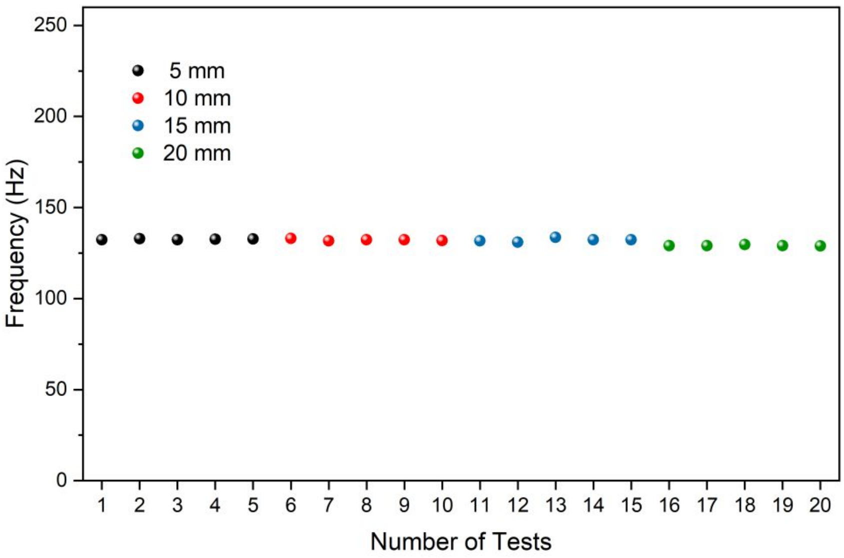

Figure S8. Eigenfrequencies of the magnetized string at stretching distances of 5 mm, 10 mm, 15 mm, and 20 mm, respectively.

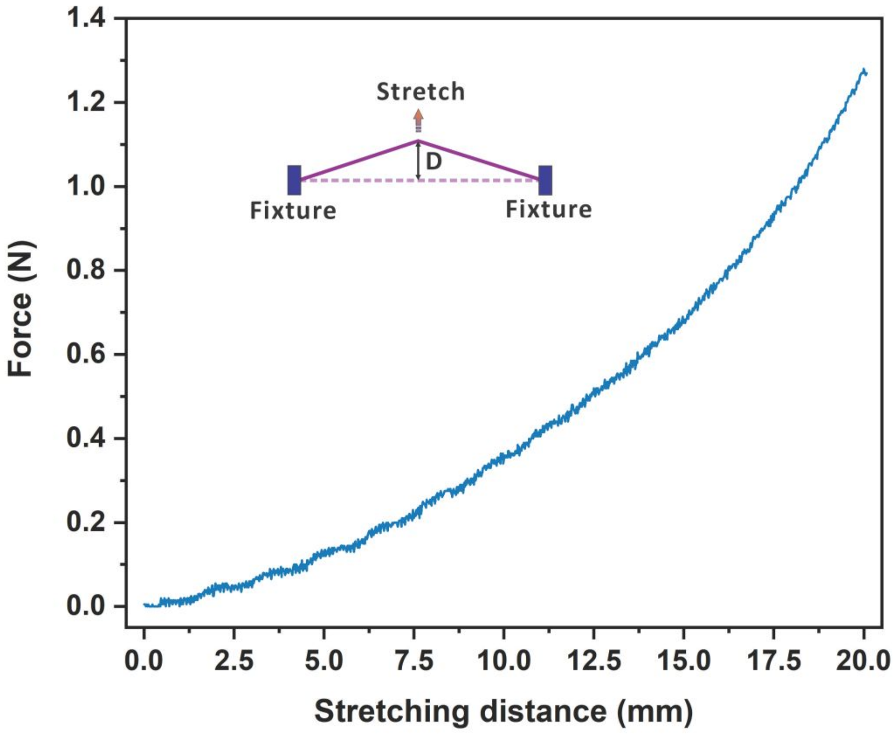

Figure S9. Relationship between the applied force and stretching distance of a typical flexible string from its equilibrium position. In the diagram, “D” indicates the stretching distance.

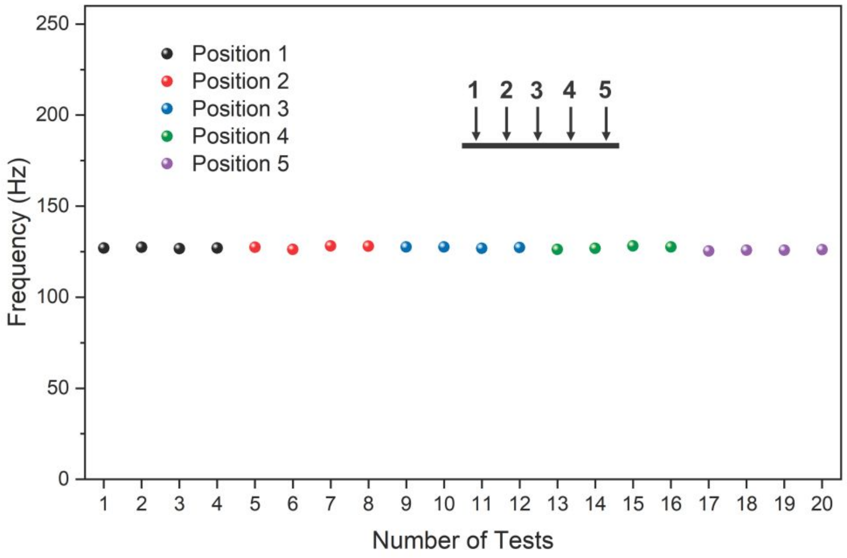

Figure S10. Eigenfrequencies of the magnetized string at different plucking positions. The plot shows the frequency stability when each position was deformed for 5 times, respectively.

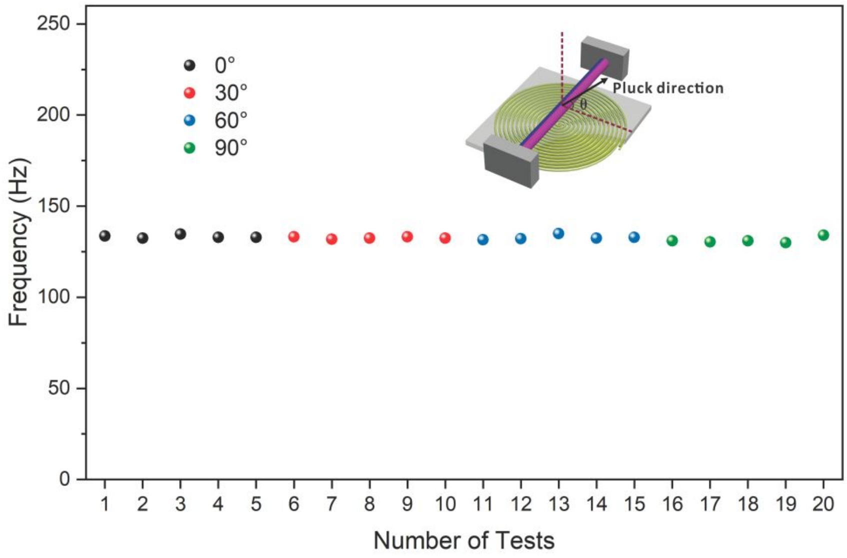

Figure S11. Eigenfrequencies of the magnetized string under different plucking angles. The value is defined by the angle between the plucking direction and the horizontal orientation.

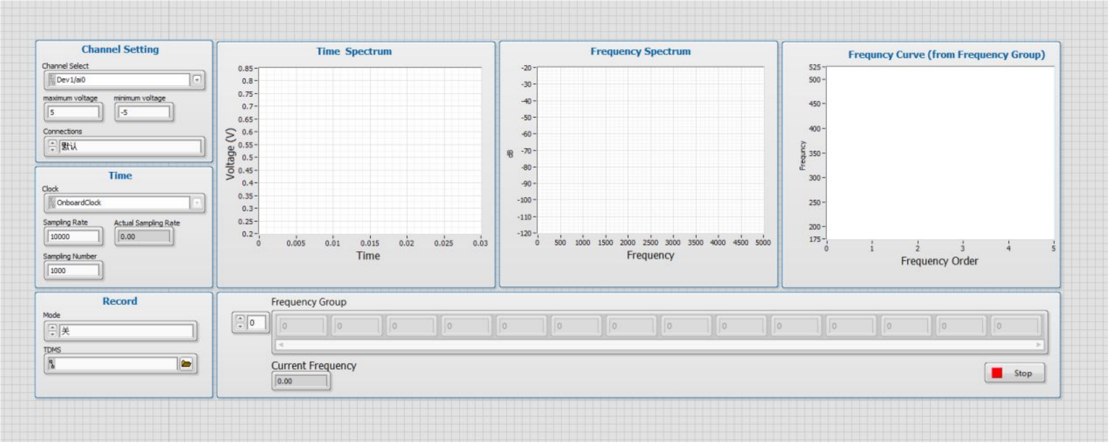

Figure S12. LabVIEW script interface.

The script is designed to record the induced current and the corresponding frequency spectra during the vibration of magnetized strings. The eigenfrequency can be directly extracted from the frequency spectra in real time.

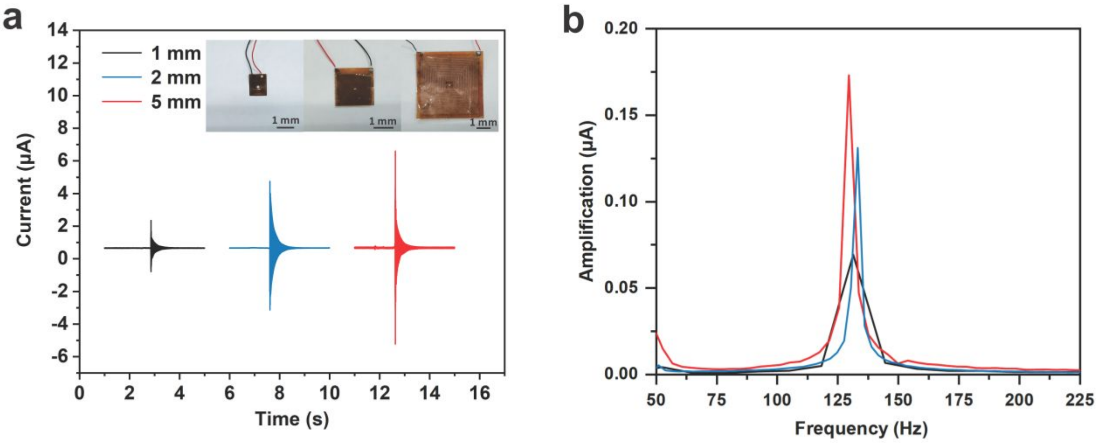

Figure S13. (a) Induced currents generated by the same magnetized string assembled with coil layer of different sizes, and (b) the corresponding frequency spectra. The dimension of the coil is 1 mm × 1 mm, 2 mm× 2 mm, and 5 mm× 5 mm, respectively.

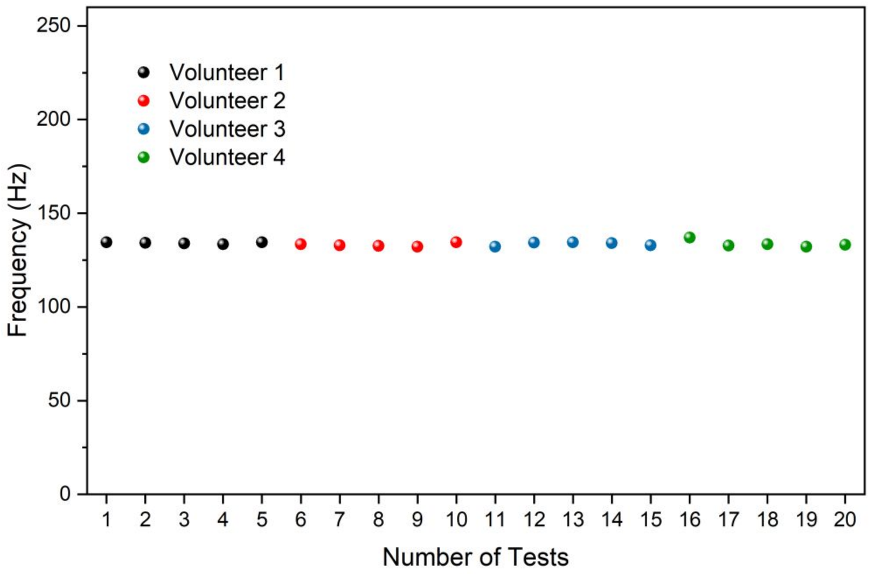

Figure S14. Eigenfrequencies of the magnetized string when manually plucked by four volunteers. Each volunteer plucked the magnetized string for 5 times to evaluate the reliability of the sensing mechanism.

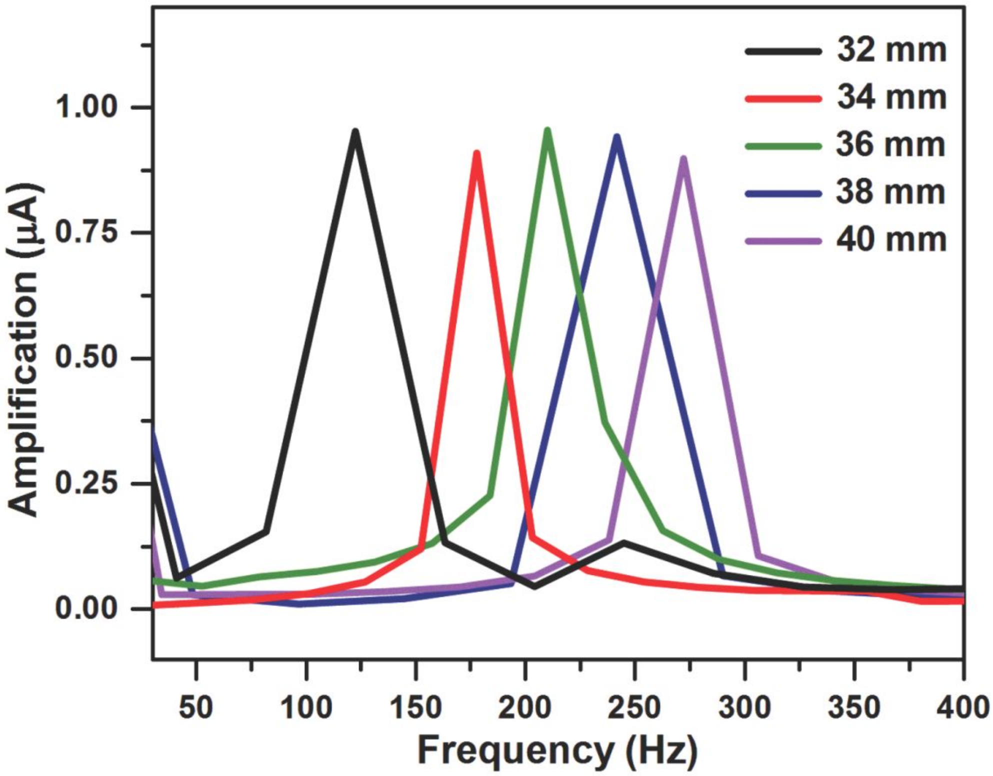

Figure S15. Frequency spectra corresponding to the induced current signals of the magnetized string at different tensile lengths of 32 mm, 34 mm, 36 mm, 38 mm, and 40 mm. The results show that the vibration eigenfrequency can be regulated via changing the tension (tensile length) within the string.

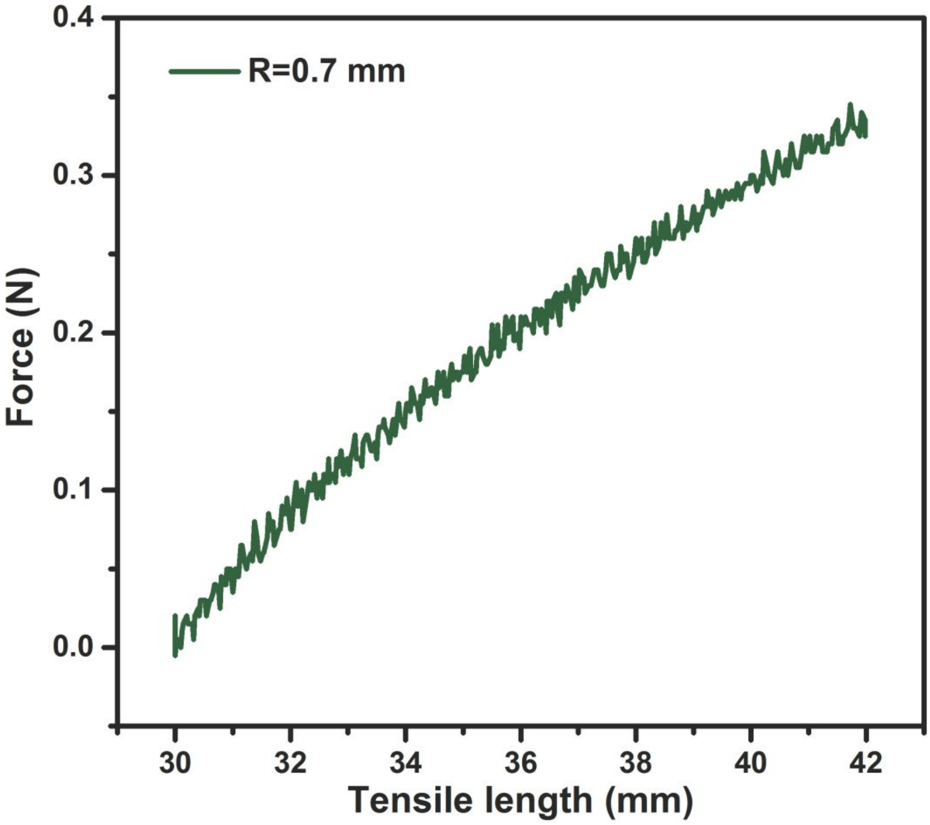

Figure S16. Recorded tension during the stretching of a magnetized string with an initial length of 30 mm. The radius of the magnetized string was 0.7 mm.

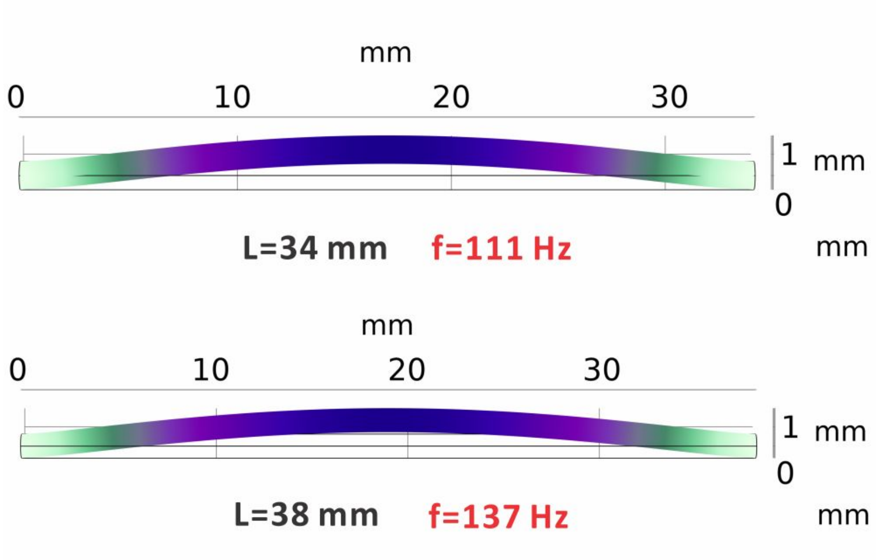

Figure S17. Simulated eigenfrequencies of the magnetized string at tensile lengths of 34 mm and 38 mm.

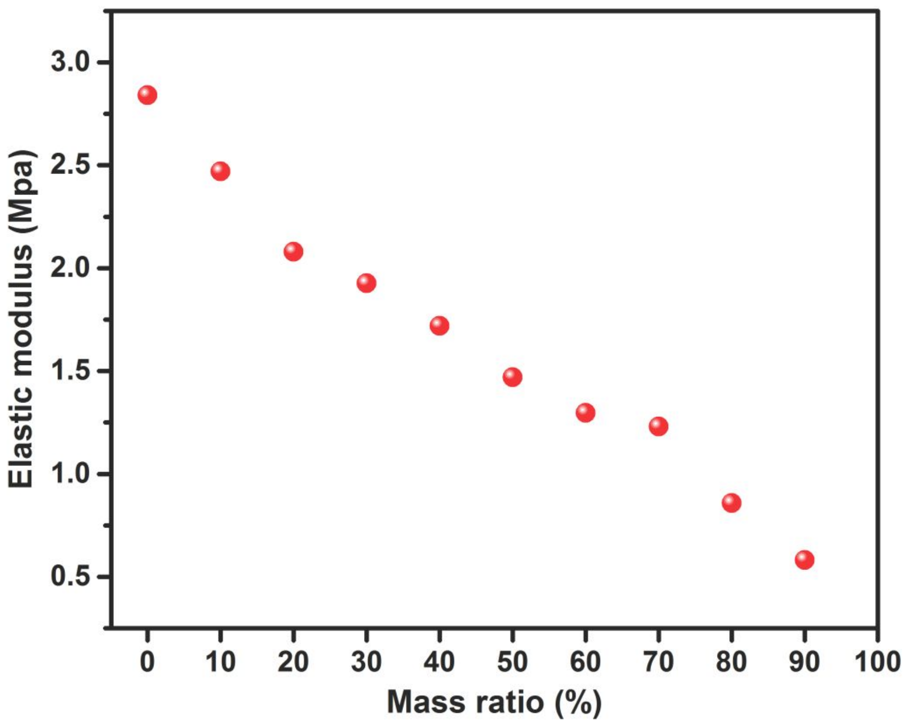

Figure S18. Elastic modulus of magnetized strings with different Ecoflex contents from 0% to 90%.

The mass ratio of silicone polymer and NdFeB particles is fixed at 1:1, while the mass ratio of PDMS and Ecoflex is regulated. The elastic modulus continuously decreases with a higher mass ratio of Ecoflex content.

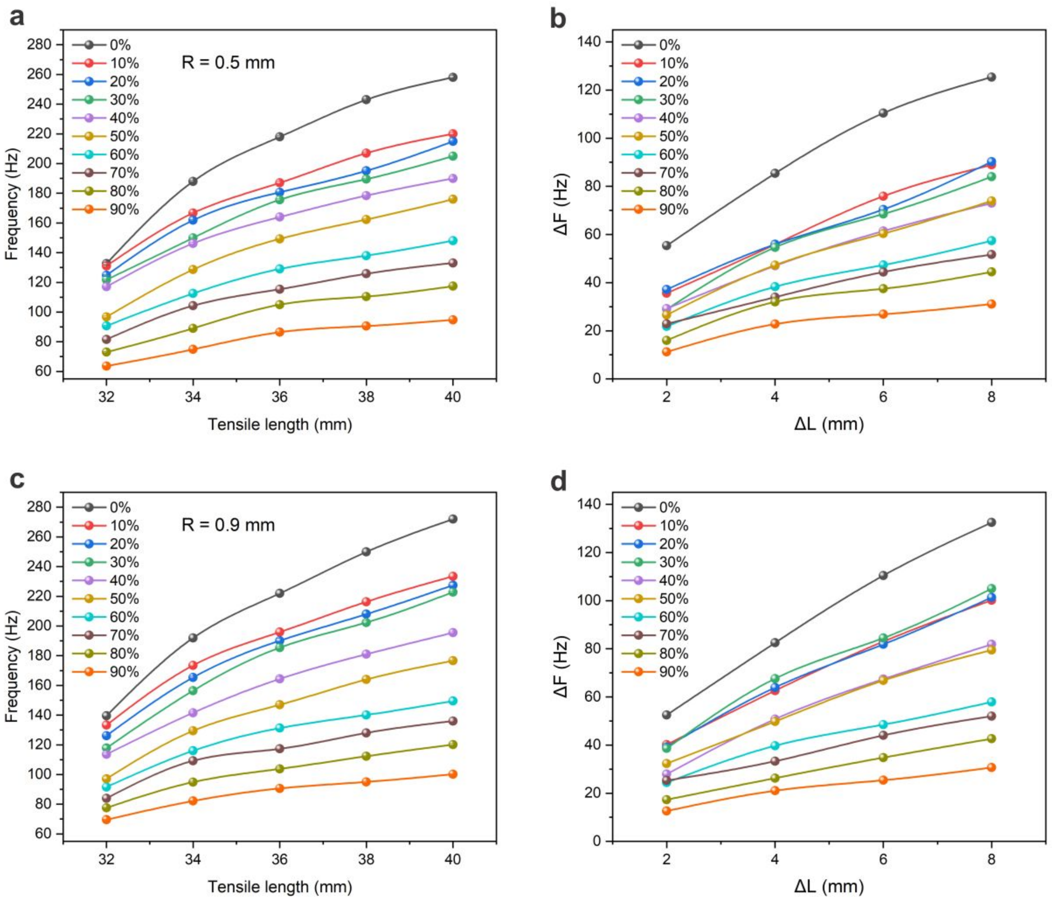

Figure S19. (a) Eigenfrequencies of magnetized strings with radius of 0.5 mm at different tensile lengths and different Ecoflex contents. (b) Effect of change in tensile length (ΔL) on change in eigenfrequency (ΔF) for magnetized strings with radius of 0.5 mm. (c) Eigenfrequencies of magnetized strings with radius of 0.9 mm at different tensile lengths and different Ecoflex contents. (d) Effect of change in tensile length (ΔL) on change in eigenfrequency (ΔF) for magnetized strings with radius of 0.9 mm.

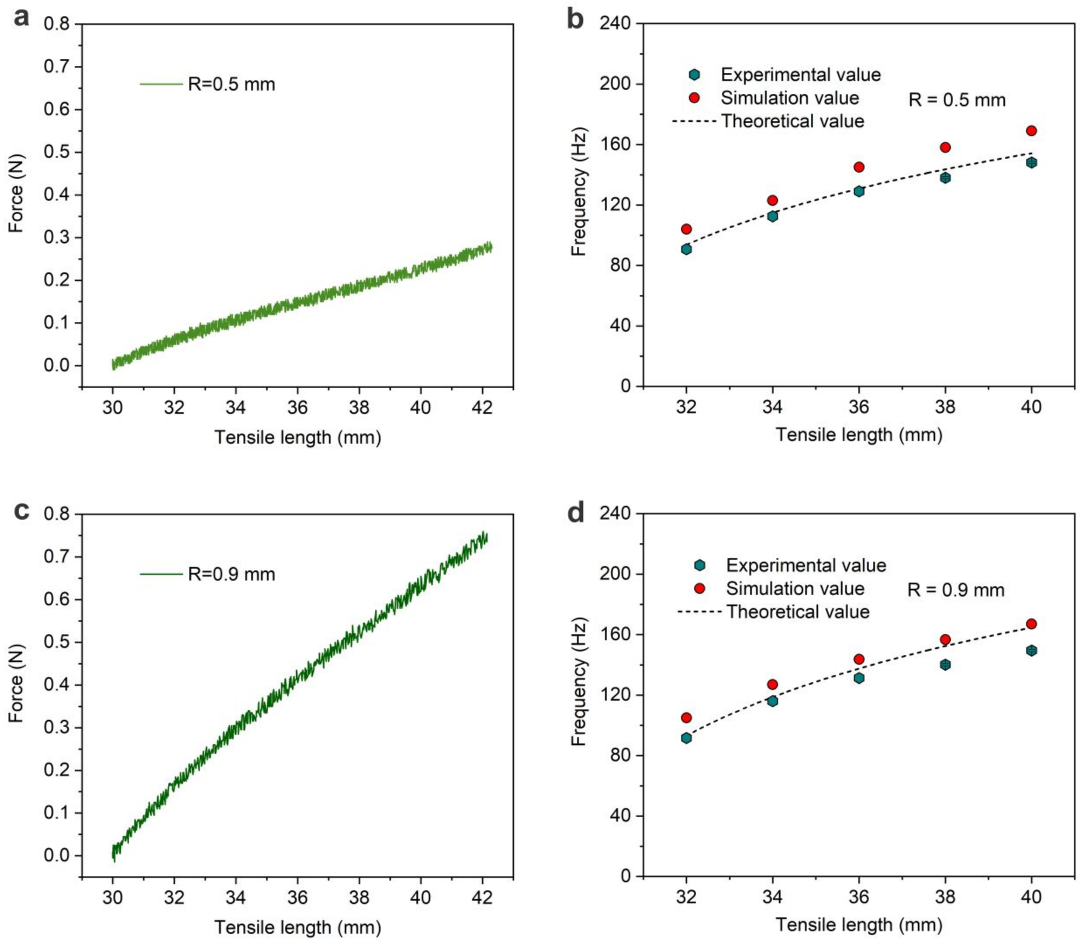

Figure S20. (a) Tension during stretching of a magnetized string with an initial length of 30 mm and radius of 0.5 mm. (b) Comparison of the experimental value, simulation value and theoretical value of string eigenfrequencies with radius of 0.5 mm. (c) Tension during stretching of a magnetized string with an initial length of 30 mm and radius of 0.9 mm. (d) Comparison of the experimental value, simulation value and theoretical value of string eigenfrequencies with radius of 0.9 mm. Here, the magnetized string under investigation was prepared in a typical mass ratio of Mp:Me:Mn at 2:3:5.

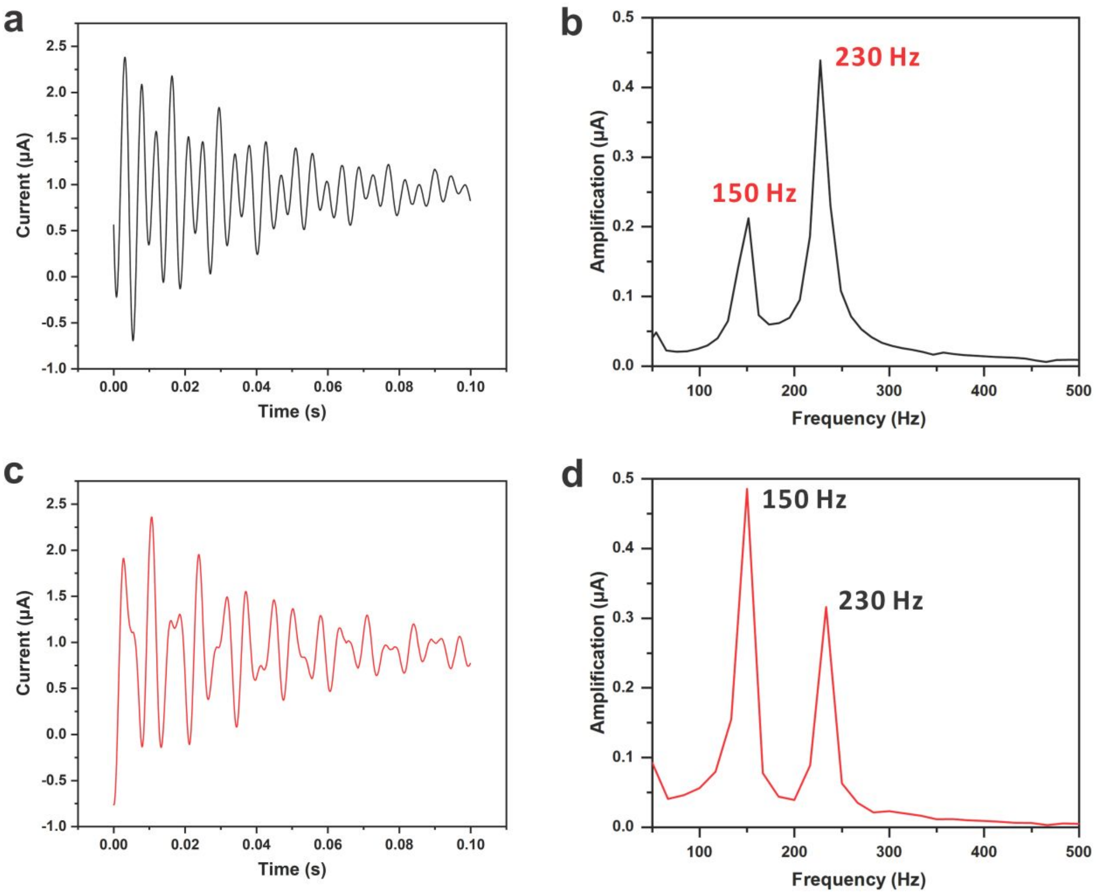

Figure S21. (a) The current signal and (b) the corresponding frequency spectra acquired by the program when the amplitude of the 230 Hz magnetized string vibration is larger. (c) The current signal and (d) the corresponding frequency spectra acquired by the program when the amplitude of the 150 Hz magnetized string vibration is larger.

Supplementary Note 1. Theoretical eigenfrequency of the magnetized string.

According to the string vibration model, the equation for the eigenfrequency (f) of a tensioned string can be described as:[1]

$$f = \frac{1}{2L}\sqrt{\frac{T}{\mu}} (1)$$

where L is the effective length, T is the tension, μ is the linear density of the string. As shown in **Figure S16**, the tension can be approximately described by a linear fitting function as:

$$T = 25.9L - 0.73434 (2)$$

The mass (m) of the selected magnetized string was measured as ~0.0929 g, and the line density can be described as:

$$\mu = \frac{m}{L} (3)$$

substituting Eq. (2) and Eq. (3) into Eq. (1), we would get:

$$f = \sqrt{69700 - \frac{1975}{L}} (4)$$

According to Eq. (4), the theoretical eigenfrequency can be obtained and plotted for comparison with the experimental and simulation value.

Supplementary Note 2. Effect of radii on string eigenfrequencyies.

According to the string vibration model, the equation for the eigenfrequency (*f*) of a tensioned string can be described as Eq. (1). For the tension (*T*), it can be described by the following equation:

$$T = \mathrm{E}\frac{\Delta L}{L_0}\pi R^2 (5)$$

where E is the elastic modulus of the string, ΔL is the length variation, L_0 is the initial length of the string, and R is the radius of the string.

For the line density of the string μ, it can be described by the following equation:

$$\mu = \pi R^2 (6)$$

where ρ is the density of the string. Substituting Eq. (5) and Eq. (6) into Eq. (1), we would get:

$$f = \frac{1}{2L}\sqrt{\frac{E\Delta L}{L_0\rho}} (7)$$

According to Eq. (7), for the same L_0 and ΔL, the effect of string radius on the eigenfrequency can be ignored. The modulus and tensile length variation would be the main parameters to define the regulation capability of the eigenfrequency.

Supplementary Videos.

Supplementary Video 1. Vibration process of a magnetized string. The vibration process of a magnetized string was recorded by a high-speed camera with a frame rate of 5000 fps. The initial length of the magnetized string was 30 mm, and the tensile length was 36 mm. The resulting video was sped down by 25X for detailed analysis (i.e. 1 s in the video equals 40 ms in real time).

Supplementary Video 2. The process of outputting four commands using four magnetized strings. Four magnetized strings with different eigenfrequencies were assembled on a coil. When the magnetized string was plucked, the induced electrical signal was processed by a LabVIEW script through a preamplifier and a DAQ (Data Acquisition) and the eigenfrequency of the signal was obtained. Different eigenfrequencies are related with different numbers that have been pre-defined by the programme interface.

Supplementary Video 3. Password input and unlocking process using four magnetized strings. The preset password for the system was defined as 2341. When the magnetized strings were plucked in the order of S2, S3, S4 and S1, the corresponding numbers 2, 3, 4, and 1 were delivered into the system. If the input password matched the preset password, the system can be unlocked. The video shows a successful unlocking process in real-time.

Supplementary Video 4. Robotic hand controlled by five magnetized strings. A robotic hand with 5 PWM servomotors can be controlled by 5 magnetized strings with different eigenfrequencies. We programmed 5 different frequencies corresponding to 5 PWM servomotors through the LabVIEW interface. When the magnetized strings S1, S2, S3, S4 and S5 were plucked, the robot's fingers were straightened in sequence.

Supplementary Video 5. The sensing process of the pressed position on a single magnetized string. When the position P1 was pressed and the magnetized string was plucked, the corresponding eigenfrequency was captured by the LabVIEW script to lighten the corresponding light. Similarly, when the positions of P2 and P3 were pressed and the magnetized string was plucked, the corresponding lamps were addressed. The video shows the process in real-time when the positions were pressed by human finger and the plucking was performed manually.

Supplementary Video 6. Robotic hand controlled by bending the wrist. The video shows the control of robotic finger bending based on the eigenfrequency change of the string that is attached on the human wrist. When the degree of wrist bending was different, the tensile length of magnetized string was different, and the eigenfrequency could be changed accordingly. Each bending angle range corresponds to a range of eigenfrequencies for identification. We programmed four different frequency ranges which are related with 4 different bending angles of the robot's fingers. Therefore, the degree of wrist bending can be applied to control the bending degree of the robot's fingers.

References

1. Perov, P.; Johnson, W.; Perova-Mello, N., The physics of guitar string vibrations. *Am. J. Phys.* **2016,** *84*, 38-43.
